# Sufu limits sepsis-induced lung inflammation via regulating phase separation of TRAF6

**DOI:** 10.7150/thno.83676

**Published:** 2023-06-26

**Authors:** Yehua Li, Jiayin Peng, Yuanxin Xia, Chenyu Pan, Yu Li, Weijie Gu, Jia Wang, Chaoxiong Wang, Yuang Wang, Jiawen Song, Xuan Zhou, Liya Ma, Yiao Jiang, Biao Liu, Qiongni Feng, Wenjia Wang, Shi Jiao, Liwei An, Dianfan Li, Zhaocai Zhou, Yun Zhao

**Affiliations:** 1 College of Life Sciences, Northwest Normal University, Lanzhou, Gansu 730070, P. R. China.; 2 State Key Laboratory of Cell Biology, Shanghai Institute of Biochemistry and Cell Biology, Center for Excellence in Molecular Cell Science, Chinese Academy of Sciences, Shanghai 200031, P. R. China.; 3 University of Chinese Academy of Sciences, Beijing 100049, P. R. China.; 4 Center for Clinical Research and Translational Medicine, Yangpu Hospital, Tongji University School of Medicine, Shanghai 200090, P. R. China.; 5 State Key Laboratory of Genetic Engineering, School of Life Sciences, Zhongshan Hospital, Fudan University, Shanghai 200438, P. R. China.; 6 Department of Medical Ultrasound, Tongji University Cancer Center, Shanghai Tenth People's Hospital, Shanghai 200072, P. R. China.; 7 School of Life Science and Technology, ShanghaiTech University, Shanghai 201210, P. R. China.; 8 Key Laboratory of Systems Health Science of Zhejiang Province, School of Life Science, Hangzhou Institute for Advanced Study, University of Chinese Academy of Sciences, Hangzhou 310024, P. R. China

**Keywords:** Sufu, TRAF6, Sepsis, Phase separation, inflammatory response

## Abstract

**Rationale:** Sepsis is a potentially life-threatening condition caused by the body's response to a severe infection. Although the identification of multiple pathways involved in inflammation, tissue damage and aberrant healing during sepsis, there remain unmet needs for the development of new therapeutic strategies essential to prevent the reoccurrence of infection and organ injuries.

**Methods:** Expression of Suppressor of Fused (Sufu) was evaluated by qRT-PCR, western blotting, and immunofluorescence in murine lung and peritoneal macrophages. The significance of Sufu expression in prognosis was assessed by Kaplan-Meier survival analysis. The GFP-TRAF6-expressing stable cell line (GFP-TRAF6 Blue cells) were constructed to evaluate phase separation of TRAF6. Phase separation of TRAF6 and the roles of Sufu in repressing TRAF6 droplet aggregation were analyzed by co-immunoprecipitation, immunofluorescence, Native-PAGE, FRAP and *in vitro* assays using purified proteins. The effects of Sufu on sepsis-induced lung inflammation were evaluated by cell function assays, LPS-induced septic shock model and polymicrobial sepsis-CLP mice model.

**Results:** We found that Sufu expression is reduced in early response to lipopolysaccharide (LPS)-induced acute inflammation in murine lung and peritoneal macrophages. Deletion of Sufu aggravated LPS-induced and CLP (cecal ligation puncture)-induced lung injury and lethality in mice, and augmented LPS-induced proinflammatory gene expression in cultured macrophages. In addition, we identified the role of Sufu as a negative regulator of the Toll-Like Receptor (TLR)-triggered inflammatory response. We further demonstrated that Sufu directly interacts with TRAF6, thereby preventing oligomerization and autoubiquitination of TRAF6. Importantly, TRAF6 underwent phase separation during LPS-induced inflammation, which is essential for subsequent ubiquitination activation and NF-κB activity. Sufu inhibits the phase-separated TRAF6 droplet formation, preventing NF-κB activation upon LPS stimulation. In a septic shock model, TRAF6 depletion rescued the augmented inflammatory phenotype in mice with myeloid cell-specific deletion of Sufu.

**Conclusions:** These findings implicated Sufu as an important inhibitor of TRAF6 in sepsis and suggest that therapeutics targeting Sufu-TRAF6 may greatly benefit the treatment of sepsis.

## Introduction

Sepsis, generally caused by dysregulated host responses to infection, is associated with acute organ dysfunction and a high risk of death 1. In sepsis, immune responses are dysregulated and fail to return to homeostasis, leading to tissue damage and ultimately organ failure. Despite the identification of multiple pathways involved in inflammation, tissue damage and aberrant healing during sepsis, there remain unmet needs for the development of new therapeutic strategies essential to prevent the reoccurrence of infection and organ injuries 2.

Hedgehog (Hh) signaling plays multiple essential roles in embryonic development, stem cell maintenance, and tissue homeostasis in metazoan from invertebrates to vertebrates. Hh signal cascade is initiated by the binding of Hh ligands to Patched 1 (Ptch1), relieving Ptch1's inhibitory effect on the signal transducer, Smoothened (SMO). The ensuing regulatory circuit permits transcriptional activation of GLI (terminal transcription factors of the Hh signaling) 3, 4. Multiple studies have revealed that Suppressor of Fused (Sufu) is a key negative regulator of the Hh signaling pathway. Sufu primarily functions as a tumor suppressor factor by interacting with and inhibiting GLIs in a Hh-dependent manner 5, 6. Sufu has also been identified to be a dual regulator integrating Hh and Wnt signals in early embryo development and human cancers 7, 8. In addition, Sufu was found to mediate EMT and Wnt/β-catenin activation in Hh-independent manner 9. A variety of cancer types are associated with the abnormally activated Hh signaling pathway in human 10-12. Although Hh signaling has been intensely studied in the context of cancer, its role in modulation of the immune response has only become evident in recent studies. The Hh signaling pathway is known to function in adult tissue repair and inflammatory response 13-15. For example, *PTCH1*-mutated tumors had higher proportions of CD8^+^ T cells, activated NK cells, and M1 type macrophage infiltration 16. Knockdown of *Gli1* exhibit increased intestinal inflammatory response to DSS with significant up-regulation of the IL-23 pathway. However, the potential function and regulatory mechanisms of Hh in sepsis remain unclarified.

Sepsis is usually initiated by activation of innate immunity, in which Toll-like receptors (TLRs) play essential roles 17. TLRs, especially TLR4 on the cell membrane, recognizes the presence of lipopolysaccharide (LPS) and activates an inflammatory response 18, 19. Dimerization of TLR4 leads to activation of NF-κB signaling, which increases the transcription of proinflammatory cytokines, resulting in inflammation and tissue damage 19. Tumor necrosis factor receptor (TNFR)-associated factor 6 (TRAF6) is thought to act downstream of multiple receptor families with immunoregulatory functions, including the TLR family 20-23. TRAF6 exerts indispensable functions in a wide array of physiological and pathological processes, in particular various aspects of innate and adaptive immunity, inflammation, and tissue homeostasis 24-27. The TRAF6 protein contains an N-terminal zinc-binding domain (containing a RING finger followed by several zinc fingers) and a C-terminal TRAF domain (consisting of a coiled-coil domain known as the TRAF-N domain and a highly conserved TRAF-C domain). It has been revealed that the N-terminal domain is essential for its E3 ubiquitin ligase activity, while the TRAF domain permits self-association and interactions with receptors and other signaling proteins 28. More work has identified mechanisms of contextual specificity for TRAF6, involving both regulatory protein interactions and direct removal of K63-linked polyubiquitin chains 29-33.

Many cellular receptors aggregate upon ligand binding to form dimers, trimers, or oligomers 34-36. Biomolecular condensates formed by liquid-liquid phase-separation (LLPS) of soluble, aggregated proteins have been studied extensively. Recent study demonstrated the LLPS of cGAS and STING in antiviral immunity 37, 38. It is worthy to investigate whether other kind of phase separation happens and plays an important role in immune response. Several studies have revealed the importance of TRAF6 self-association for its activation in signaling transduction and both N-terminal and C-terminal regions of TRAF6 contribute to its homo-oligomerization 39-41. However, it remains unknown whether TRAF6 also undergoes LLPS in inflammatory signaling and how the process was regulated.

In this study, we investigated the interplay between sepsis and Hh signaling, and uncovered a novel functional mechanism of TRAF6: that TRAF6 undergoes phase separation for signaling in LPS-induced innate immunity, which is essential for subsequent autoubiquitination and NF-κB activation. We further demonstrated that Sufu restrains the activity of TRAF6 via interfering its phase separation. Our study discovered an anti-inflammatory role of Sufu in sepsis-induced lung inflammatory response and identifies the Sufu-TRAF6 axis as a potential therapeutic target for the treatment of sepsis.

## Materials and Methods

### Mice

Male (6-8 weeks old) mice of the following genotypes and strains were used: LyzM-Cre (The Jackson Laboratory, Number 004781, maintained on a C57BL/6 background) and Sufu(flox/flox) (generously provided by Dr. Chi-Chung Hui lab and maintained on C57BL/6 background) 42. For induction of septic shock models, 6-8 weeks old mice were injected intraperitoneally with LPS (5 mg/kg). For PEMs, mice were given intraperitoneal injection of thioglycolate broth to elicit peritoneal macrophages. All experiments represent a minimum of n = 3 mice. All animal studies were performed in accordance with the guidelines of the Institutional Animal Care and Use Committee of the Center for Excellence in Molecular Cell Science, Shanghai Institute of Biochemistry and Cell Biology, Chinese Academy of Sciences. Animals were housed in SPF conditions, kept under standard conditions with a 12 h day/night cycle and access to food and water ad libitum.

### Cell culture

HEK-293FT were purchased from Thermo Fisher Scientific (R700-07). HEK-BlueTM-hTLR4-cells were obtained by co-transfection of the human TLR4, MD-2 and CD14 co-receptor genes, and an inducible secreted embryonic alkaline phosphatase (SEAP) reporter gene into HEK-293 cells (InvivoGen, hkb-hTLR4, and 16I16-MM). HEK-293FT and HEK-BlueTM-hTLR4-cells were maintained in Dulbecco's modified Eagle's medium (DMEM). BMDMs were generated after culture for a week with complete RPMI 1640 medium plus G-MSF. All cell culture media were supplemented with 10% fetal bovine serum and 1% penicillin, streptomycin, or Normocin^TM^.

### Reagents

LPS (*E. coli,* serotype 055:B5), anti-Flag antibody (F3165), anti-Myc antibody (M4439) and anti-β-actin antibody (A2228) were purchased from Sigma. Poly(I:C), R-848 and ODN 1585 were from Enzo Life Sciences. DAPI (D1306) were purchased from Invitrogen. Antibody to p-IKKβ (2694), IKKβ (2678), p-JNK (4668), JNK (9252), p-ERK (4376), ERK (4695), p-IκBα (9246), IκBα (4812), MyD88 (4283), HA (3724), TRAF6 (4743; immunofluorescence analysis), and Sufu (2522) were purchased from Cell Signaling. Anti-His (sc-8036), anti-TRAF6 (sc-8409) (immunoprecipitation and immunoblot analysis), anti-IRAK1 (sc-5288) and anti-IRAK4 (sc-374349) were purchased from Santa Cruz. Goat anti-mouse IgG (31430) and goat anti-rabbit IgG (31460) were purchased from Thermo Fisher Scientific. C25-140 (HY-120934) was purchased from MedChemExpress.

### Plasmids

Mammalian expression vectors for Flag-MyD88, Flag-IRAK1, Flag-IRAK4 and Flag-TRAF6 have been described 41, 43. Sufu were cloned into pCDNA-3.1 with sequence encoding a Myc-tag. All lentiviral plasmids were constructed in a GFP-tagged-p23 vector. Human wild-type Sufu were cloned into a His-TEV-pET28a vector for hexahistidine (His_6_)-tagged recombinant proteins. TRAF6-N (amino acid 1-347) domain and TRAF6-C domain of TRAF6 (amino acid 348-522) was cloned into GFP-pET28a or pET28a vector, respectively, for purification proteins of GFP-tagged TRAF6-N and TRAF6-C.

### Lentivirus infection and generation of stable cell lines

To produce lentiviral particles, HEK-BlueTM-hTLR4-cells (70%-80% confluence) in a 10 cm dish were co-transfected with 8 μg GFP-p23-TRAF6, 6 μg psPAX2 and 2 μg pMD2.G. The supernatant containing viral particles were harvested twice at 48 and 72 h after transfection, filtered through Millex-GP Filter Unit (0.45 μm pore size, Millipore), and stored at -80 °C until use. To infect HEK-BlueTM-hTLR4-cells with lentivirus, cells were cultured in medium containing lentivirus and 1 μg/mL polybrene (Sigma). To increase the efficiency, infected cells were subjected to several days of puromycin selection. The cells were allowed to recover for at least 3 days before performing subsequent experiments.

### Transfection and reporter assay

Transient transfection of cells was performed using Lipofectamine 2000 from Invitrogen, according to the manufacturer's instructions. HEK-293FT cells were transfected with plasmid encoding the NF-κB luciferase reporter and luciferase activities were determined using the Dual-Luciferase assay system (Promega).

### Quantitative reverse transcription-PCR (qRT-PCR) analysis

qRT-PCR was performed on an Applied Biosystems StepTwo Real-Time PCR System (Applied Biosystems) and results were calculated by the comparative cycling threshold (Ct) quantization method. Real-time PCR Master Mix (TOYOBO) was used to detect and quantify the expression of the target gene. The gene encoding *Gapdh* was used as an internal control. The primers used as listed in Table S1.

### ELISA

Serum and PEMs culture supernatants were collected and assayed for cytokines. Cytokine production was measured by ELISA of TNFα (SMTA00B for mouse) and IL-6 (SM600B for mouse) according to the protocol of the manufacturer (R&D Systems).

### Immunoprecipitation and immunoblot analysis

For immunoprecipitation experiments, whole-cell extracts were prepared after transfection or stimulation, and were incubated overnight with the appropriate antibodies (identified above), together with Protein A/G beads (Santa Cruz). Beads were then washed three times with lysis buffer, and immunoprecipitated were eluted with sodium dodecyl sulfate (SDS) loading buffer and resolved by PAGE. The proteins were transferred to a polyvinylidene difluoride (PVDF) membrane and were further incubated with the appropriate antibodies (identified above).

### Native-PAGE

Blue cells or 293FT cells were treated as indicated, and then rinsed twice with ice-cold PBS and suspended in 0.5 mL RIPA buffer, containing proteinase inhibitor followed by sonication. Cell lysates were centrifuged at 1,000 g for 10 min at 4°C and the supernatants were precleared with flag-tagged beads for 2 h at 4°C followed by washing three times with RIPA buffer. The beads were washed with ice-cold PBS two times. Protein samples were mixed with 4X loading buffer (without SDS) and loaded into precast Native-PAGE gels (without SDS) for western blot.

### Protein expression and purification

The recombinant proteins used in this manuscript were all expressed in E. coli strain BL21 (DE3). All cells were lysed with lysis buffer (20 mM Tris, pH 7.5, 500 mM NaCl and 10% glycerol). For His- or MBP-tagged proteins, Ni^2+^-NTA agarose (GE Healthcare) or MBP beads (NEB) were used for affinity chromatography according to the manufacturer's instructions. Each sample was further purified by size-exclusion chromatography in buffer containing 20 mM HEPES, pH 7.5, and 100 mM NaCl.

### Immunofluorescence

Cells were seeded onto 35-mm glass bottom dishes and grown for 24 h before the indicated treatment. Cells were washed once with PBS and fixed in 4% formaldehyde in PBS for 15 min, followed by permeabilization with Triton X-100 (0.05%) in PBS for 5 min. Cells were then blocked with PBS containing BSA (5%) for 1 h and then incubated with primary antibodies for 1 h. After three separate washes, cells were incubated with a secondary antibody for 1 h. For tissues staining, freshly dissected mouse lung was fixed in 4% paraformaldehyde at 4 °C for 1 h and washed in PBS for three times. Then, the tissues were dehydrated in 30% sucrose overnight at 4 °C and embedded in OCT. 10 μm cryosections were obtained and air-dried afterwards at room temperature. For staining, dried sections were washed in PBS and then blocked with 1% BSA and 0.1% Triton X-100 in PBS for 30 min at room temperature. Sections were incubated overnight at 4 °C with the primary antibodies: anti-TRAF6 antibody (Santa, sc-8409) and anti-F4/80 antibody (abcam, ab6640). Primary antibodies were detected using fluorescent conjugated secondary antibodies (Invitrogen, Alexa Fluor 488 and Alexa Fluor 555). Cells and sections were stained with DAPI (4′,6-diamidino-2-phenylindole) and mounted with Aqua-Ploy/mount (Polysciences). Images were captured using an Olympus FV3000 confocal microscope. ImageJ software was used to analyze the collected image.

### Preparation of RNA-seq libraries and sequencing

For PEMs, mice were given intraperitoneal injection of thioglycolate broth to elicit peritoneal macrophages. PEM were isolated from WT and Sufu-cKO mice. Isolated PEMs were seeded and cultured at 37 °C, 5% CO_2_ for 48 h. Then, cells were treated with LPS for 3 h, respectively. Cells were collected directly into TRIzol and RNA was extracted using a QIAGEN RNeasy Mini Kit according to the manufacturer's instructions. The integrity of the isolated RNA was verified using a Bioanalyzer (Agilent 2100). 10 ng of high-quality RNA (RNA integrity number, RIN > 8) was used to produce cDNA libraries using the VAHTS mRNA-seq V3 Library Prep Kit for Illumina (Vazyme) according to the manufacturer's instructions and sequenced on an Illumina NextSeq sequencer.

### FACS

For sorting lung macrophages and neutrophil, mice were anesthetized with ketamine and xylazine. To prepare single-cell suspensions, total lung tissue was harvested, digested in 1640 medium containing 2 mg/mL collagenase A and 20 U/mL DNase I, and filtered through a 40-µm cell strainer. FACS was performed using FACS Aria III (BD Biosciences) to isolate macrophages and neutrophil.

For lung macrophages analysis, mice were injected intraperitoneally with LPS (5 mg/kg) or equal volumes of PBS. 24 h later, mice were anesthetized, and lung single-cell suspensions were obtained as above. For bronchoalveolar lavage (BAL) cells analysis, BAL was performed with 10 serial lavages of 1 mL PBS containing 5 mM EDTA from LPS- and CLP-induced WT and Sufu-cKO mice. BAL cells were washed twice in PBS. Cells were incubated with primary antibodies for 60 min on ice, washed with cold medium. Flow cytometric analysis was performed using LSR Fortessa (BD Biosciences). FlowJo software was used for data analysis.

### Fluorescence Recovery After Photobleaching (FRAP) assay

Cells were cultured on 35 mm No.1.5 glass-bottomed dishes. All FRAP assays were performed on Leica TCS SP8 STED 3X microscope equipped with a 10031.4 NA HC PL APO CS2 oil immersion objective and operated with the LAS-X imaging software. The region of interest was photobleached and the recovery of fluorescence intensity within the region of interest was obtained for each experiment. Intensity recovery curves were normalized and corrected for photobleaching 44. The recovery curves were fit to the following expression by GraphPad:

Y(t)=A· (1-*e*^ (*τ* ·*t* ))

Where A is the end-value of the recovered intensity, t is the fitted parameter and t is the time after the bleaching pulse.

### Phase separation assay

Phase separation assay was performed as described previously with modifications 45. The purified Emerald-tagged TRAF6 (1-347aa) were assembled by diluting the protein from a high salt-containing storage buffer to a physiological buffer (20 mM Tris PH 7.5, 150 mM NaCl, 0.1 mM PMSF) or physiological buffer with 10% polyethylene glycol. Samples were prepared on High Performance No.1.5 18 × 18 mm glass coverslips (Schott) and were imaged within 30 min after drop assembly with a DeltaVision Elite imaging system.

### Survival studies in sepsis

All experimental mice were sex- and age-matched. For survival studies of LPS-induced sepsis, adult WT and Sufu-cKO mice were intraperitoneally injected with a dose at 15 mg/kg LPS. For survival studies of CLP-induced sepsis, mice were anesthetized and a 1-1.5 cm midline incision was made. About 50% of the cecum was ligated and the cecum was punctured twice with a 21-gauge needle. A small amount of feces was extruded from the hole to ensure patency. The abdominal incision was closed by applying sample running sutures. Then, pre-warmed PBS was injected subcutaneously. Mice were monitored every 6 h and the death time was recorded.

### TRAF6 siRNA transfection in lung

Small interfering RNA (siRNA) duplex oligonucleotides were purchased from GenePharma. The sequences of TRAF6 siRNA and negative control (NC) duplex were 5′-GAGAACAGAUGCCUAAUCATT-3′ and 5′-UAAGGCUAUGAAGAGAUAC-3′, respectively. The siRNA-mediated knockdown of TRAF6 in PEMs were performed by transfecting cells with 10 nmol/L siRNA at 70% confluence using the protocol provided by the manufacturer. For the *in vivo* knockdown of TRAF6, 8-week-old Sufu-cKO mice were injected with Entranster-*in vivo* reagent (Engreen Biosystem) carrying TRAF6 siRNA through the caudal vein. Successful transfection of TRAF6 siRNA was confirmed by TRAF6 qRT-PCR and western blotting of lung homogenates.

### Determination of vascular permeability in lung

For vascular permeability studies of endotoxemia-induced lungs, adult WT and Sufu-cKO mice were intraperitoneally injected with PBS or 5 mg/kg LPS for 24 h and Evans blue (MedChemExpress) was injected to the caudal vein of mice at a dose of 30 mg/kg for 40 min before euthanasia and lung collections. Lung tissues were then perfused with ice-cold PBS containing 0.6 mmol/L EDTA, and dried with tissue papers. Left lung tissues were weighed, taken pictures and snap frozen in liquid nitrogen. The right lung tissues were homogenized in 0.5 mL PBS and incubated with 1 mL formamide (Yeasen) for 18 h at 60 °C followed by centrifuged for 20 min at 12000 rpm. The Evans blue absorption (A) of tissue supernatants was verified at 620 nm. The Evans blue index was calculated as ratio between corrected A_620_ and the weight of lung tissues. Corrected A_620_ = observed A_620_ - (1.1649 × A_740_ + 0.004).

### Lung histopathology

To determine histopathologic lung injury scores in lungs, fixed lung tissues were embedded in paraffin, sectioned for HE staining following standard protocols as previously described. Then lung tissues were sectioned at 5 μm thickness. Sections were stained with hematoxylin and eosin to evaluate the degree of lung injury using light microscopy. The degree of injury was scored based on the presence of exudates, hyperemia/congestion, neutrophilic infiltration, inter-alveolar hemorrhage/debris, and cellular hyperplasia. The severity of injury was judged according to the following criteria: no injury = 0; injury to 25% of the field = 1; injury to 50% of the field = 2; injury to 75% of the field = 3; and diffuse injury = 4. Lung injury score was calculated as the sum of scores from 4 different views of the lung tissue section of each mouse (3 mice/group).

### Statistical analysis

Estimated sample size for a planned comparison of two independent means using a two-tailed test was undertaken using an on-line calculator and the SAS statistical software package (version 9.1.3). Data are expressed as means ± SEM for continuous variables and as frequencies and proportions for categorical variables. Continuous data were compared using Student's *t* test. For correlation, the Spearman rank correlation was used. Survival curves were calculated according to the Kaplan-Meier method; survival analysis was performed using the log rank test. *p* values of < 0.05 were considered significant.

## Results

### Dynamic expression of Sufu in response to inflammatory stimuli

**Figure 1:**
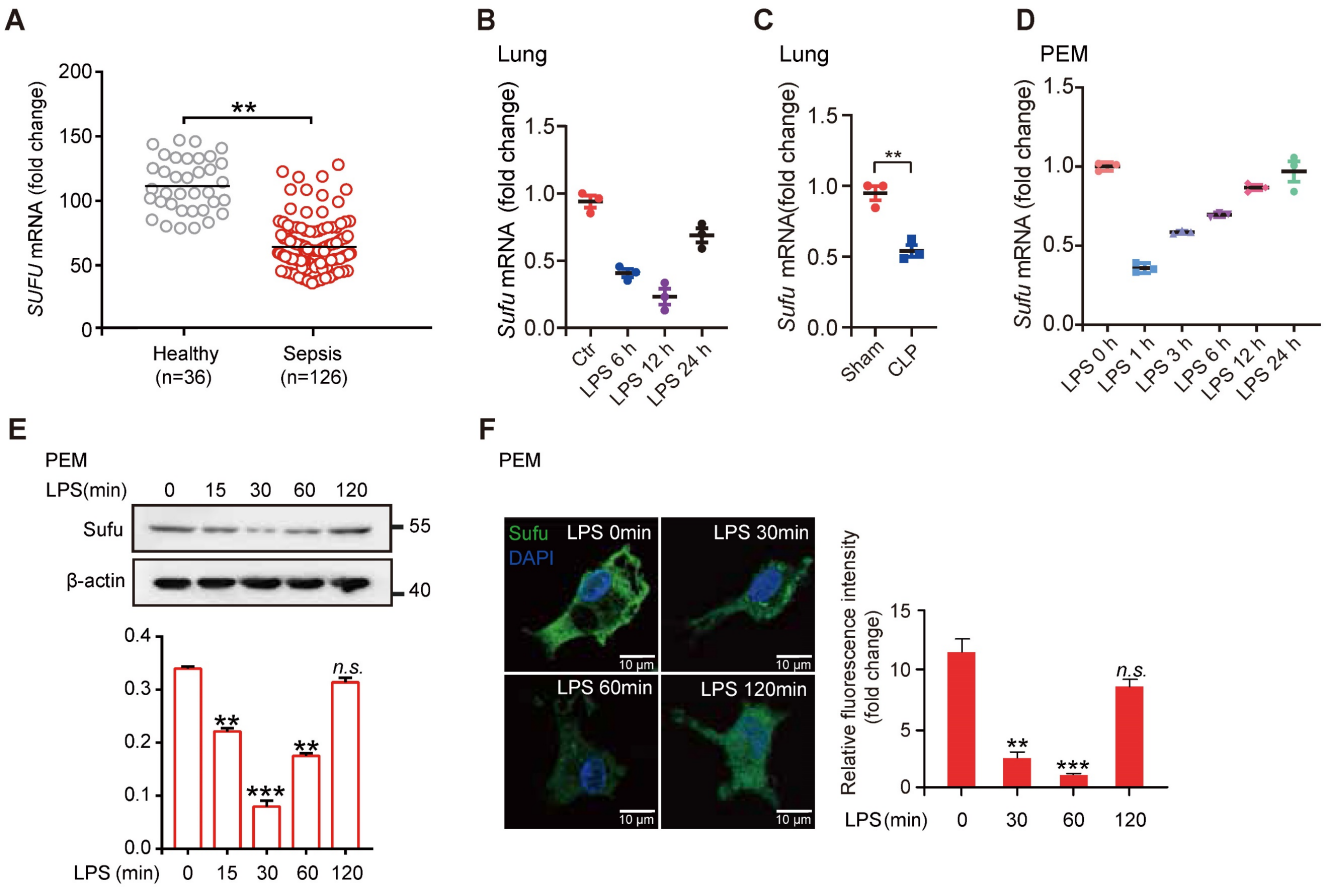
** LPS inhibits Sufu expression in mice lungs and PEMs.** (**A**) Scatter plot for *Sufu* mRNA levels from human sepsis data set GSE54514. Means ± SEM. **p < 0.01. (**B**) Quantitative analysis of *Sufu* mRNA levels in the lungs of wild-type (WT) mice after LPS (5 mg/kg) challenge at indicated times by qRT-PCR. Results are presented relative to those of the control gene *Gapdh* and normalized to 0 h. (**C**) Quantitative analysis of *Sufu* mRNA level in the lungs of WT mice after CLP by qRT-PCR. Results are presented relative to those of the control gene *Gapdh*. (**D**) Quantitative analysis of *Sufu* mRNA levels in PEMs after LPS (100 ng/mL) challenge at indicated times by qRT-PCR. Results are presented relative to those of the control gene *Gapdh* and normalized to 0 h. (**E**) Immunoblot analysis of Sufu and β-actin (loading control) in PEMs after LPS challenge (100 ng/mL) at the indicated time points (horizontal axis). Bar graph indicated the Sufu protein levels normalized to β-actin. Means ± SEM (n = 3). **p < 0.01. ***p < 0.001. n.s., no significance. (**F**) Immunostaining for Sufu in PEMs after LPS challenge (100 ng/mL) at indicated time points. **Left**, representative images of Sufu staining in PEMs. Scale bar, 50 μm. **Right**, quantification of fluorescence intensity of Sufu in PEMs. Means ± SEM (n = 5). **p < 0.01. ***p < 0.001. n.s., no significance.

To evaluate a potential role for Hh signaling in sepsis, we first examined the expression levels of essential genes of this pathway and the levels of known genes involved in inflammatory responses from peritoneal exudate macrophages (PEMs) and bone marrow derived macrophages (BMDMs) after lipopolysaccharide (LPS) stimulation (GEO data sets, PEMs: GSE2002 and BMDMs: GSE53986). We observed that the expression of Sufu was significantly decreased in both kinds of cells after LPS treatment, whereas no significant differences in expressions of other major Hh components in PEMs were observed (Figure S1A). We also assessed the correlation between the expression levels of* SUFU* and infectious diseases by analyzing clinical data from the GEO database. We observed that *SUFU* mRNA was significantly down-regulated in peripheral blood samples of patients with sepsis (GEO data sets GSE54514; Figure 1A). Meanwhile, we analyzed gene expression data from two independent published datasets (GDS1276: Inflammatory lung injury and mechanical ventilation and GDS1239: Inflammatory lung injury and effect of simvastatin). Both of which suggested that Sufu was downregulate after LPS treatment (Figure S1B). To verify the above observations, we utilized LPS-challenged septic shock model by intraperitoneally injecting mice with 5mg/kg LPS. The results showed that *Sufu* mRNA was decreased in the lungs of the challenged mice after LPS stimulation (Figure 1B). Similar results were obtained in CLP-induced polymicrobial sepsis model (Figure 1C). We next treated mouse PEMs with LPS, and analyzed the kinetics of *Sufu* transcription upon LPS stimulation, as well as the expression of inflammatory cytokine genes, such as* Tnfα* and* Il-6*. Consistent with previous discoveries 41, 43, 46, we also found that a rapid increase of *Tnfα* mRNA level within the first hour, and then decreased to basal level by 24 h (Figure S1C). However, the expression of *Il-6* was delayed by several hours relative to the expression of *Tnfα*, peaking at 6 h post-stimulation (Figure S1D). Interestingly, the expression level of* Sufu* quickly dropped by about half within 1 h, and then gradually recovered to the basal levels in 24 h (Figure 1D). We then further evaluated the cellular protein level of Sufu in response to LPS. The protein level of Sufu decreased within 30 min after LPS stimulation, and gradually recovered to the basal level by 120 min (Figure 1E). Correspondingly, a semiquantitative immunofluorescence assay performed in isolated PEMs also revealed dynamic expression of Sufu during LPS challenge (Figure 1F). We also evaluated the transcriptional levels of the Hh pathway components, such as *Smo* and *Ptch1* (Figure S1E-F). We found that the transcription level of *Ptch1* showed little response to LPS stimulation, while *Smo* mRNA level displayed an opposite fluctuation to that of Sufu. Similar to the results for LPS stimulation, the expression of Sufu level also reduced by other TLR ligands, such as poly (I:C) (a ligand of TLR3), R-848 (a ligand of TLR7) and ODN1585 (a ligand of TLR9) (Figure S1G-L), indicating that Sufu might generally respond to TLR signaling. Taken together, these results uncovered that the expression of Sufu changes in response to inflammatory stimulation in macrophages, indicating that Sufu may play an important role in innate immune response.

### Deletion of Sufu in myeloid cells aggravates septic shock

**Figure 2:**
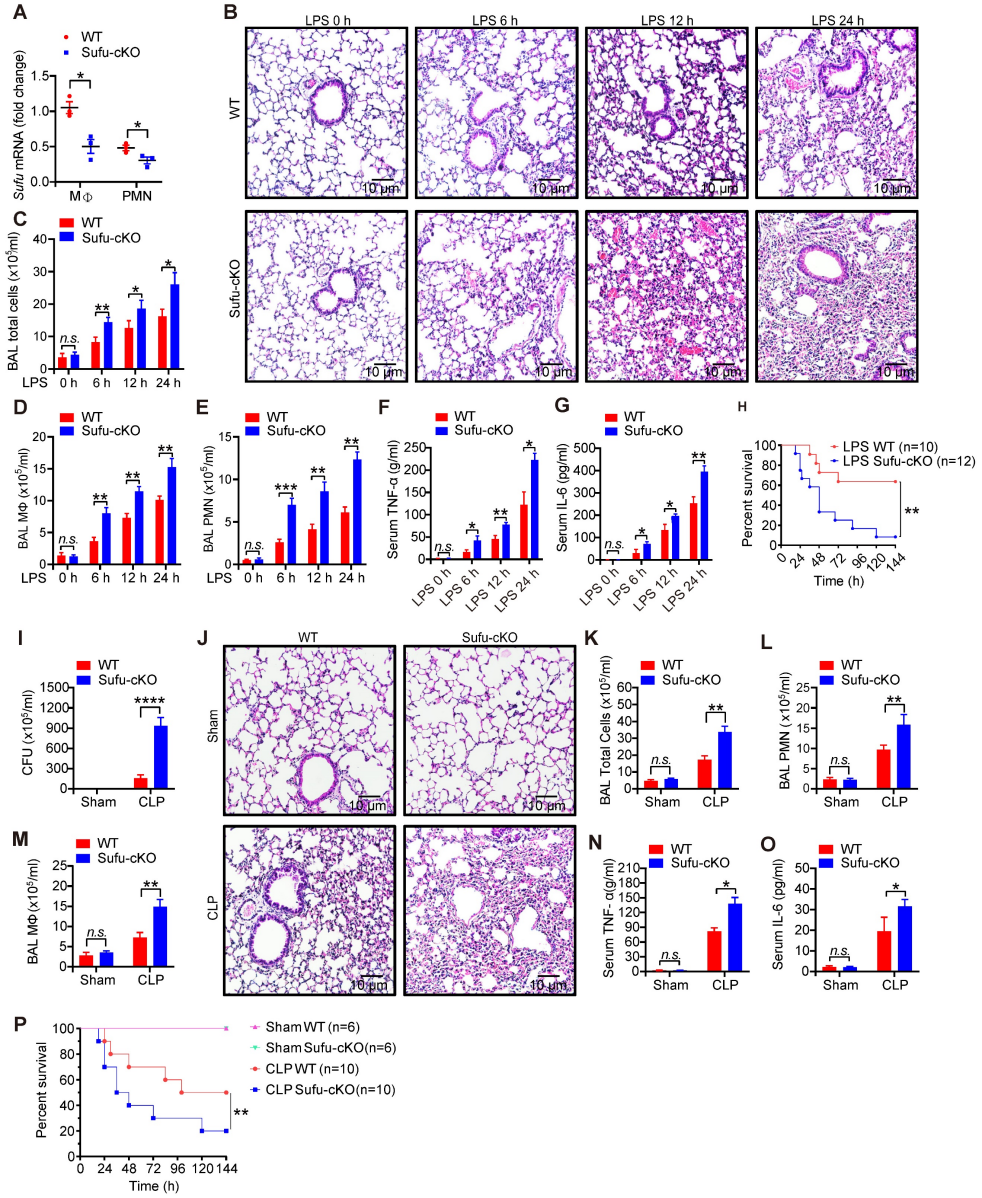
** Myeloid-specific ablation of Sufu aggravates LPS- and CLP-induced septic shock.** (**A**) The expression of *Sufu* measured by qRT-PCR in lung macrophages and neutrophils isolated from WT and Sufu-cKO mice. Means ± SEM (n = 3). *p < 0.05. (**B**) Hematoxylin and eosin (H&E) staining of lung tissue sections from WT and Sufu-cKO mice injected intraperitoneally with LPS (5 mg/kg) and euthanized at indicated times. Scale bar, 10 μm. (**C-E**) WT and Sufu-cKO mice were injected intraperitoneally with LPS (5 mg/kg) at indicated times, and BALF were collected to count the total number of cells (**C**), macrophages (**D**) and neutrophils (**E**) by flow cytometry. Means ± SEM (n = 3). *p < 0.05. **p < 0.01. ***p < 0.001. n.s., no significance. (**F and G**) Levels of TNFα (**F**) and IL-6 (**G**) in serums of WT and Sufu-cKO mice after LPS challenge at indicated times. Means ± SEM (n = 3). *p < 0.05. **p < 0.01. n.s., no significance. (**H**) Survival curves of WT and Sufu-cKO mice following LPS challenge (15 mg/kg). Log-rank (Mantel-Cox) test (n = 10 and 12). **p < 0.01. (**I**) Bacterial colony-forming units (CFU) in the peritoneal cavity of WT or Sufu-cKO mice 20 h after CLP. Means ± SEM (n = 5). ****p < 0.0001. (**J**) Representative hematoxylin and eosin staining of the lungs from WT or Sufu-cKO mice 20 h after CLP. Scale bar, 10 μm. (**K, L and M**) Numbers of total cells (**K**), macrophages (**M**) and neutrophils (**L**) in the BALF of WT or Sufu-cKO mice 20 h after CLP. Means ± SEM (n = 3). **p < 0.01. n.s., no significance. (**N and O**) Serum levels of TNFα (**N**) and IL-6 (**O**) 20h after CLP in WT or Sufu-cKO mice. Means ± SEM (n = 3). *p < 0.05. n.s., no significance. (**P**) Survival curves of WT and Sufu-cKO mice following CLP. Log-rank (Mantel-Cox) test (n = 6-10). **p < 0.01.

To verify the potential role of Sufu in innate immunity *in vivo*, we generated myeloid-specific Sufu knockout mice (referred to as Sufu-cKO mice) by crossing Sufu (flox/flox) mice with LysM-driven (LysM-Cre) transgenic mice. Myeloid cell-specific Sufu deficiency showed a significant decreased the expression of Sufu in Sufu-cKO mice compared to WT mice (Figure 2A and Figure S2A). Next, we examined the effects of Sufu deletion on the response to septic shock in WT and Sufu-cKO mice using the LPS-challenged septic shock model. Histological analysis showed morphological evidence of lung injury, including severe disruption of alveolar walls and widespread alveolar wall thickenings in association with edema and infiltration by mononuclear cells, all of which were exacerbated in LPS-challenged Sufu-cKO mice compared to WT mice (Figure 2B). Compared with WT mice, Sufu-cKO mice showed substantially more total cells, macrophages, and neutrophils in bronchoalveolar lavage (BAL) (Figure 2C-E). The production of proinflammatory cytokines (TNFα and IL-6) was greatly elevated in the serum of Sufu-cKO mice after LPS stimulation as compared with WT mice (Figure 2F-G). However, the survival rate of Sufu-cKO mice was significantly lower than that of WT mice after LPS challenge (Figure 2H). These data indicate that the myeloid-specific deletion of Sufu aggravated LPS-induced septic shock, suggesting that Sufu negatively regulates innate inflammatory responses.

To further determine whether this observation could be replicated in a clinically relevant sepsis model, we induced polymicrobial sepsis in WT and Sufu-cKO mice by CLP. The number of peritoneal aerobic bacteria was increased in Sufu-cKO mice compared with WT mice at 20 h after CLP (Figure 2I). CLP-induced immune cell infiltrations in lungs were enhanced in Sufu-cKO mice, which was consistent with LPS treatment (Figure 2J). We next assessed the influx of macrophages and neutrophils in the BAL of WT and Sufu-cKO mice after CLP. No differences were observed between sham-exposed samples in WT mice and Sufu-cKO mice (Figure 2K-M). However, the numbers of total cells, macrophages and neutrophils in BAL were significantly increased in Sufu-cKO mice compared to WT mice after CLP (Figure 2K-M), and the serum levels of TNFα and IL-6 were significantly increased in Sufu-cKO mice (Figure 2N-O). Moreover, the CLP-challenged Sufu-cKO mice showed markedly shorter survival than CLP-challenged WT mice (Figure 2P). These results indicate that Sufu-cKO mice are more susceptible to CLP-induced peritonitis than WT mice.

### Deletion of Sufu in macrophages promotes inflammatory responses

**Figure 3:**
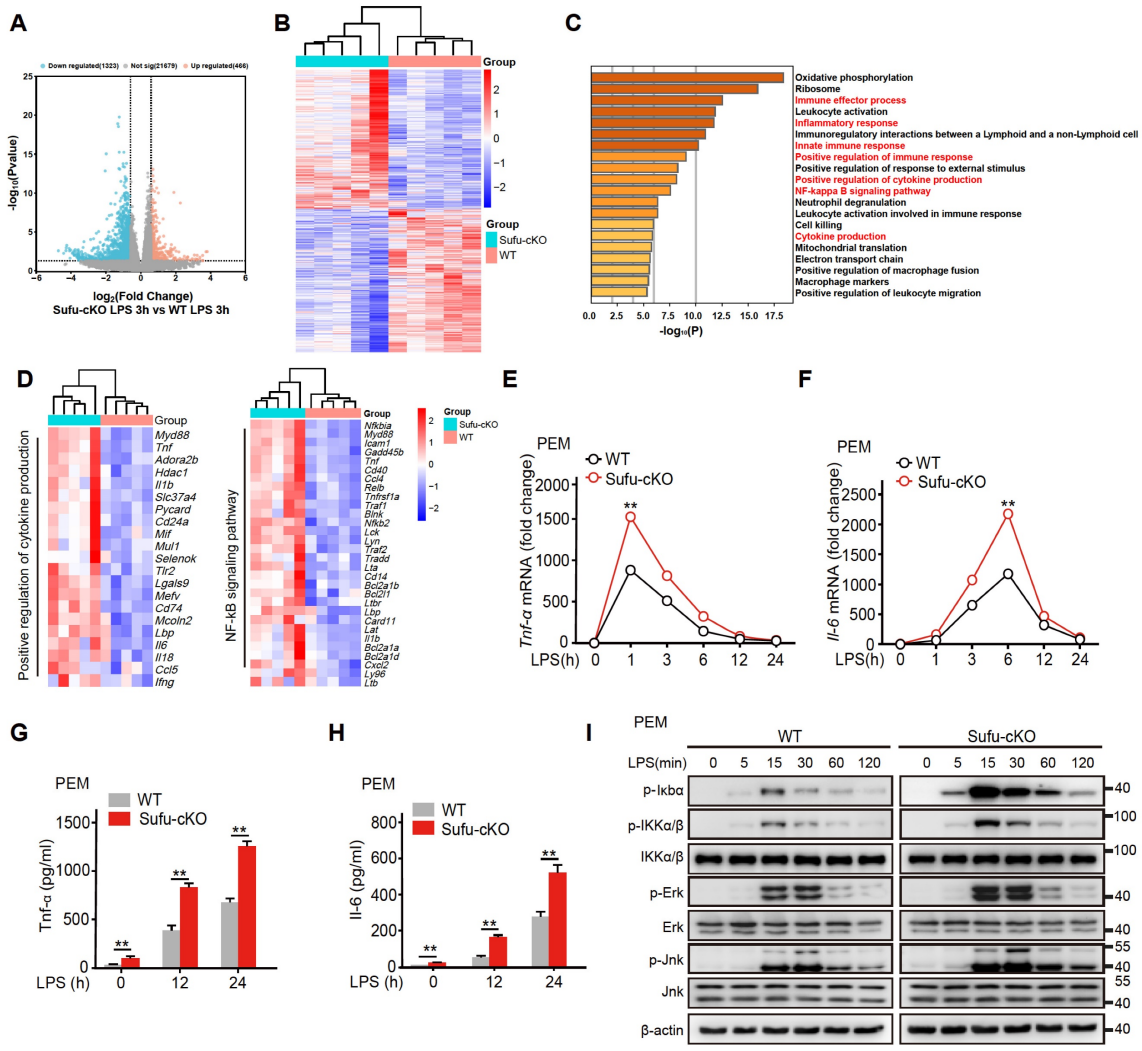
** Deletion of Sufu enhances inflammatory responses.** (**A**) Volcano plots depicting the differentially expressed genes in PEMs from WT and Sufu-cKO mice 3 h after LPS challenge. (**B**) Heatmap of RNA-seq data showing enriched genes in PEMs from WT and Sufu-cKO mice 3 h after LPS challenge. (**C**) Gene Ontology (GO) annotation in PEMs from WT and Sufu-cKO mice 3 h after LPS challenge. The enriched cytokine-receptor interaction and inflammatory response pathways were highlighted by red words. (**D**) Heatmap showing expression of select specific genes. (**E and F**) The expression of *Tnfα* (**E**) and *Il-6* (**F**) mRNA level in PEMs isolated from WT and Sufu-cKO mice at indicated time points after LPS challenge (100 ng/mL). Means ± SEM (n = 3). **p < 0.05. (**G and H**) Secretion of TNFα (**G**) and IL-6 (**H**) by PEMs at 12h and 24 h post LPS challenge (100 ng/mL). Means ± SEM (n = 3). **p < 0.05. (**I**) Immunoblot analysis of phosphorylated (p-) and total IKKα/β, Erk, Jnk, p-Iκbα, and β-actin (loading control) in lysates of PEMs derived from WT or Sufu-cKO mice challenged with LPS (100 ng/mL) and isolated at indicated time points post challenge.

To study the regulatory mechanism of Sufu in LPS-induced inflammatory signaling, we also performed RNA-seq in PEMs from WT and Sufu-cKO mice 3 h after LPS challenge. Among the differentially expressed genes (DEGs), 466 were up-regulated, and 1323 were down-regulated in WT cells compared with Sufu-cKO cells (Figure 3A). Meanwhile, we evaluated differentially expressed genes. This analysis revealed that Sufu-cKO macrophages upregulated genes for inflammatory responses (Figure 3B). To identify potential signaling pathways that were altered by Sufu deletion, differentially regulated genes were further classified by Gene Ontology (GO) analysis. GO analysis revealed that genes regulating the inflammatory response, regulation of cytokine production and NF-κB signaling were significantly up-regulated (Figure 3C). We noticed genes required for regulation of cytokine production and NF-κB signaling were significantly up-regulated (Figure 3D), suggesting that Sufu deletion in the myeloid compartment intensified macrophage pro-inflammatory response.

As mentioned above, the expression of Sufu induced by LPS exhibited a certain degree of specificity in macrophages. Next, we further examined the specific mechanism by which Sufu exerted a proinflammatory role in LPS-treated PEMs. PEMs were isolated from Sufu-cKO or WT mice and stimulated with LPS at different time points. RT-qPCR and ELISA analysis unveiled that Tnfα and Il-6 expression were significantly increased in Sufu-cKO macrophages compared with WT counterparts following LPS stimulation (Figure 3E-H). We also evaluated the regulatory effect of Sufu on acute TLR4 signaling by detecting the phosphorylation of IKKα/β, Jnk, Erk, and p-IκBα in PEMs. LPS induced significant degradation of IκBα and activation of downstream kinases (IKKα/β, Jnk and Erk) in the control group (Figure 3I). However, deletion of Sufu further enhanced TLR4 signaling transduction, as shown by increased phosphorylation of downstream kinases (IKKα/β, Jnk and Erk) (Figure 3I). We further confirmed these observations with bone marrow-derived macrophage (BMDMs) (Figure S2B). These data confirmed that deletion of Sufu enhances inflammatory responses and TLR-induced NF-κB signaling in macrophages.

### Sufu directly interacts with TRAF6 and mediates its autoubiquitination

**Figure 4:**
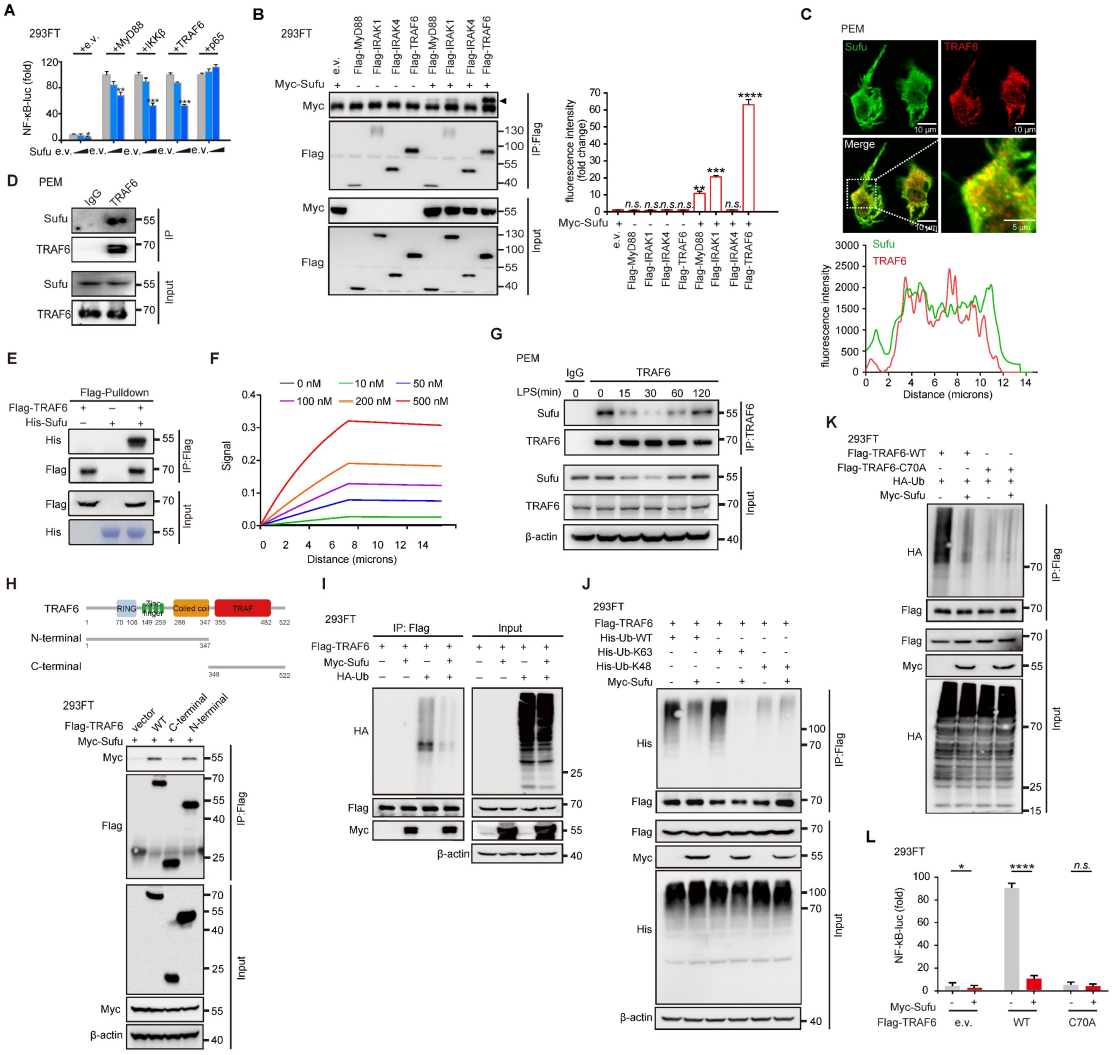
** Sufu inhibits TLR4 signaling through attenuating autoubiquitination of TRAF6.** (**A**) Luciferase activity of 293FT cells transfected with an NF-κB luciferase reporter and indicated TLR4 components, plus either empty vector or increasing dose of Sufu. Results are presented relative to those of cells transfected to express empty vector alone. Means ± SD (n = 5). *p < 0.05. **p < 0.01. ***p < 0.001. (**B**) Co-immunoprecipitation of Sufu with components of TLR4 signaling complexes. Means ± SD (n = 3). **p < 0.01. ***p < 0.001. ****p < 0.0001. n.s., no significance. (**C**) **Above**, representative image of Sufu (Green) and TRAF6 (Red) localization in PEMs. **Bottom**, line graph analysis of the fluorescence intensity showing the co-localization between Sufu and TRAF6. Scale bar, 10 μm. (**D**) Co-IP to detect the interaction between endogenous Sufu and TRAF6 in PEMs. (**E**) *In vitro* pull-down assay of His-Sufu and Flag-TRAF6. (**F**) SPR sonograms reflecting the control surface-subtracted interactions between recombinant Sufu and TRAF6. (G) Interaction between Sufu and TRAF6 in PEMs challenged with LPS (100 ng/mL) for indicated times. Data are representative of three independent experiments with similar results (**H**) **Above**, schematic drawing of TRAF6 domains and truncations used. **Bottom**, co-immunoprecipitation of Sufu with TRAF6 truncations in the lysates of 293FT cells transfected with Myc-Sufu and Flag-TRAF6 truncations. (**I**) Ubiquitination of TRAF6 in 293FT cells transduced with HA-ubiquitin (HA-Ub) and Flag-TRAF6, plus Myc-Sufu or control vector. (**J**) Ubiquitination of TRAF6 in 293FT cells transduced with Flag-TRAF6 and His-WT ubiquitin (His-Ub-WT) or mutants (His-Ub-K63/K48), plus Myc-Sufu or control vector. (**K**) Ubiquitination of TRAF6 in 293FT cells transduced with HA ubiquitin (HA-Ub) and Flag-TRAF6 or TRAF6-C70A, plus Myc-Sufu or control vector. (**L**) Luciferase activity of 293FT cells transfected with an NF-κB luciferase reporter and with either empty vector or Flag-tagged WT TRAF6 or TRAF6-C70A, plus Myc-Sufu or control vector. Means ± SD (n = 3). **p < 0.01. ****p < 0.0001. n.s., no significance.

To further investigate the regulatory mechanism of Sufu on innate immune response, we transfected 293FT cells with an NF-κB luciferase reporter and increasing doses of Sufu. Sufu strongly inhibited the activation of NF-κB in a dose-dependent manner (Figure 4A). Next, we used co-immunoprecipitation (Co-IP) to analyze the association of Sufu with a variety of TLR4 signaling pathway components, including MyD88, IRAK1, IRAK4 and TRAF6. Sufu could be immunoprecipitated together with MyD88 and IRAK1, but exhibited stronger association with TRAF6 (Figure 4B). We speculated that Sufu forms a complex with MyD88, IRAK4 and TRAF6. Following this line, we performed siRNA-mediated knock-down of Myd88, IRAK1 or IRAK4, respectively, and then detected the association between Sufu and TRAF6. The result showed that among the individual components of this complex, Sufu and TRAF6 may have a dominant interaction, as none of the knock-downs prevented Sufu and TRAF6 binding (Figure S3A). These observations were further confirmed by a semiquantitative immunofluorescence assay, which showed colocalization of endogenous Sufu and TRAF6 proteins (Figure 4C), as well as by co-immunoprecipitation of these endogenous proteins in PEMs (Figure 4D). Binding between Sufu and TRAF6 was further confirmed by *in vitro* precipitation experiments using the purified recombinant Sufu protein and the TRAF6 protein produced by the *in vitro* translation system. His-tagged Sufu was readily precipitated by Flag-tagged TRAF6, indicating a direct interaction between these two proteins (Figure 4E). Similarly, Myc-tagged Sufu directly associated with Flag-TRAF6 (Figure S3B). Furthermore, a surface plasmon resonance (BIAcore) assay revealed a dose-dependent binding between Sufu and TRAF6 (Figure 4F). Consistent with the LPS-induced expression pattern of Sufu, the interaction between Sufu and TRAF6 decreased during the first 15-30 min of LPS challenge and recovered afterwards (Figure 4G). Subsequent domain mapping revealed that the N-terminal region of TRAF6 (amino acids 1-347) was required for its interaction with Sufu and the Coiled-coil domain (amino acids 288-347) of Sufu is important for its interaction with TRAF6 (Figure 4H and Figure S3C-D). Taken together, these results indicated that Sufu directly interacts with TRAF6 and might regulate LPS-induced TLR4 signaling through such binding.

It is well studied that Lys63 (K63)-linked autoubiquitination is a key regulatory event for the activation of TRAF6 47-49. Therefore, we investigated whether Sufu affects autoubiquitination of TRAF6. We transfected 293FT cells with HA-tagged ubiquitin (HA-Ub) and Flag-tagged TRAF6 in the presence or absence of Myc-tagged Sufu, then performed immunoprecipitation experiments with anti-FLAG antibody. When expressed in cells along with HA-Ub, Flag-tagged TRAF6 that was immunoprecipitated and analyzed by immunoblot with anti-HA antibody showed high levels of ubiquitination; however, such ubiquitination of TRAF6 was substantially attenuated in the presence of Sufu (Figure 4I). Since K48-linked ubiquitination can induce degradation while K63-linked ubiquitination mainly regulates signaling transduction 50, 51, we then examined the specific ubiquitination type of TRAF6 influenced by Sufu. In contrast to WT-Ub and K63-Ub, overexpression of Sufu did not alter the ubiquitination of TRAF6 by transfecting K48-Ub, indicating that Sufu specifically inhibited the K63-linked ubiquitination of TRAF6 (Figure 4J). Meanwhile, we employed C25-140 (TRAF6-Ubc13 inhibitor) with Sufu knockout PEMs before LPS treatment. The results showed that knockout of Sufu promoted the ubiquitination of endogenous TRAF6 upon stimulation with LPS compared with control group. Importantly, such ubiquitination of TRAF6 spontaneously formed in PEMs from Sufu-cKO mice even in the absence of LPS, and LPS stimulation further promoted TRAF6 ubiquitination levels. While, C25-140 treatment strongly diminished the ubiquitination of TRAF6 in PEMs derived from WT mice and Sufu-cKO mice, respectively (Figure S3E). Additionally, overexpression of Sufu did not alter the ubiquitination of TRAF6 mutant defective of its E3 ligase activity (cysteine at position 70 was replaced by alanine, C70A) (Figure 4K), indicating that Sufu inhibited TRAF6 autoubiquitination. Consistent with these observations, NF-κB luciferase reporter assay showed that the autoubiquitination of wild type TRAF6, but not the C70A TRAF6 mutant, was considerably diminished by Sufu (Figure 4L). Taken together, these results demonstrated that Sufu inhibits K63-linked autoubiquitination of TRAF6.

### TRAF6 undergoes phase separation in response to LPS signaling

**Figure 5:**
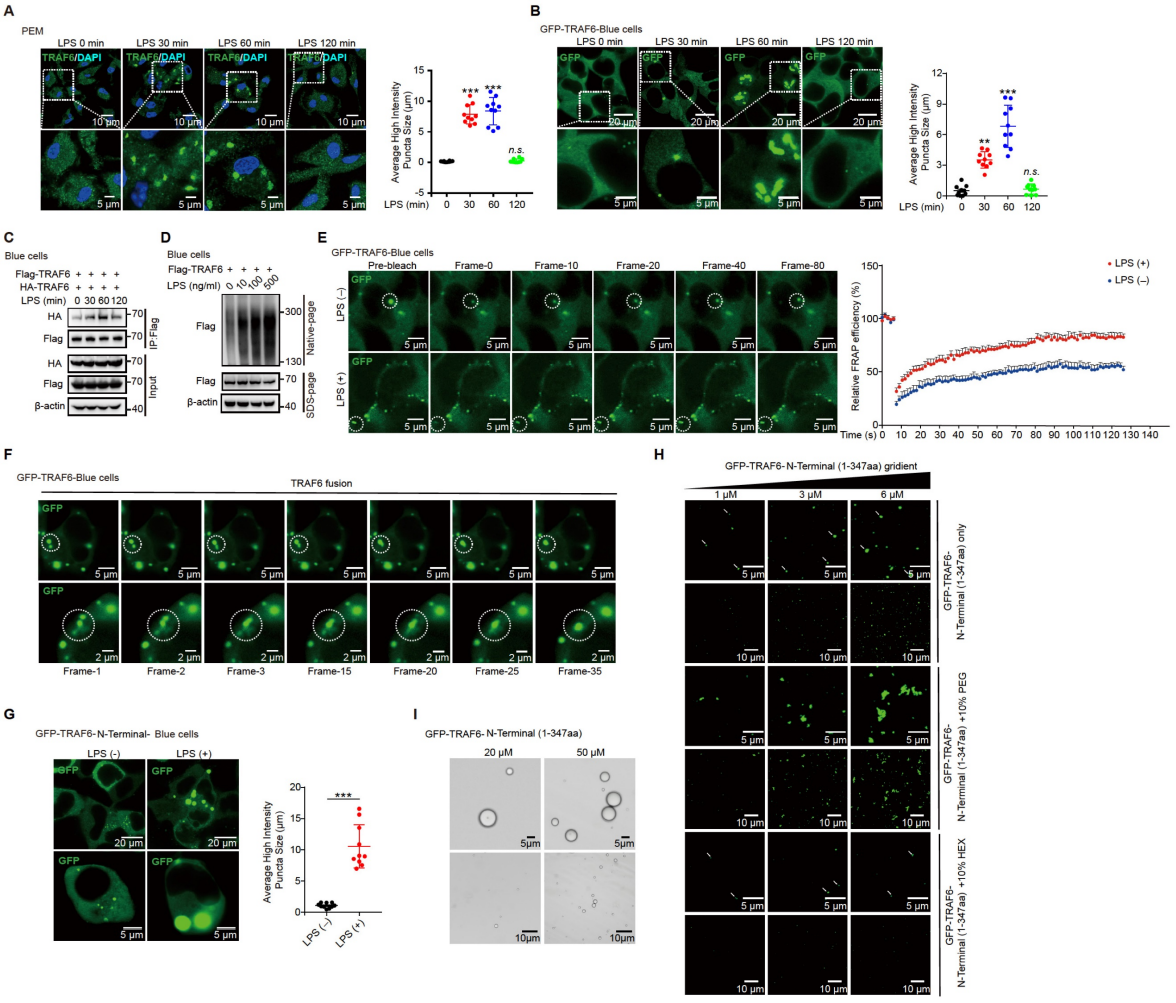
** Phase separation of TRAF6 during LPS stimulation.** (**A**) **Above**, Representative images of TRAF6 (Green) at various time post LPS challenge (100 ng/mL) in PEMs. DAPI (Blue) was used for nuclear staining. Scale bar, 10 μm. **Bottom**, magnification of the boxed region. Scale bar, 5 μm. (**B**) **Above**, Representative images of GFP (Green) at various time after challenge with LPS (100 ng/mL) in GFP-TRAF6-Blue cells. Scale bar, 20 μm. **Bottom**, magnification of the boxed region. Scale bar, 5 μm. (**C**) Co-IP of Flag-tagged TRAF6 and HA-tagged TRAF6 in Blue cells challenge with LPS for indicated number of times and assessed by immunoblot analysis with anti-Flag or anti-HA after immunoprecipitation with anti-Flag. (**D**) Self-association of TRAF6 in Blue cells transfected with Flag-tagged TRAF6 before challenged with increasing doses of LPS as indicated, and analyzed by immunoblot analysis with anti-Flag in Native-PAGE. (**E**) **Left**, Representative fluorescent recovery after photobleaching (FRAP) images of TRAF6 (Green) in GFP-TRAF6-Blue cells. Scale bar, 5 μm. White circles indicate bleached regions. For examples of fluorescence bleaching, see Videos S1 and S2. **Right**, Kinetics of TRAF6 recovery in GFP-TRAF6-Blue cells. (**F**) GFP-TRAF6-Blue cells was challenged with LPS (100 ng/mL) and imaged after 30 min. Frames were taken every 7.5 s. Scale bar, 5 μm or 2 μm. For sample fusion events of TRAF6 in living cells, see Video S3 and S4. (**G**) Representative images of N-terminal-TRAF6 with or without LPS (100 ng/mL) stimulation. Scale bar, 20 μm (**Above**) or 5 μm (**Bottom**). (**H**) Increased concentration of GFP-TRAF6-N-Terminal proteins formed liquid-like droplets that were treated with 10% PEG-8000 or 10% HEX, respectively. White arrowheads indicate liquid-like droplets. Scale bar, 5 μm or 10 μm. (**I**) GFP-TRAF6-N-Terminal phase separation with different concentrations (20 μM and 50 μM) of protein. Scale bar, 5 μm (**Above**) or 10 μm (**Bottom**).

Homo-oligomerization of TRAF6 has been reported to be important for its autoubiquitination and subsequent activation 39, 40. Multivalent interactions between proteins can promote self-association, driving phase-separated droplet assembly in the cell 52, 53. To investigate whetherTRAF6 undergoes dynamic oligomerization during TLR4 signaling, we firstly performed immunofluorescence staining of endogenous TRAF6 proteins in PEM cells stimulated with LPS. The results showed that TRAF6 was evenly distributed in the cytoplasm in the absence of LPS stimulation (Figure 5A). Surprisingly, conspicuous aggregations of TRAF6 appeared in PEMs within 30 min of LPS treatment, which peaked at 60 min and then gradually diminished at 120 min (Figure 5A). Furthermore, we overexpressed GFP-tagged TRAF6 (GFP-TRAF6) in Blue cells (a 293FT stable cell line expressing TLR4, CD14 and MD2) and constructed GFP-TRAF6-expressing stable cell line (GFP-TRAF6 Blue cells), which were used to evaluate the homo-oligomerization and possible phase separation of TRAF6. In the absence of LPS treatment, overexpression of TRAF6 resulted in a lesser extent of aggregation of TRAF6, and then exhibited a similar response to LPS treatment observed for endogenous TRAF6 proteins in PEMs (Figure 5B). Consistent with these results, a co-IP assay in 293FT cells transfected with Flag-tagged TRAF6 and HA-tagged TRAF6 showed self-association of TRAF6 in response to LPS (Figure 5C). Additionally, we treated Blue cells overexpressing Flag-tagged TRAF6 with different doses of LPS, and then used Native-PAGE to detect homo-associated proteins as polymers. Consistent with findings described above (Figure 5A-C), increased concentration of LPS promoted TRAF6-self-association in Blue cells (Figure 5D).

Next, we conducted fluorescence recovery after photobleaching (FRAP) assay to examine the association kinetics of TRAF6 in GFP-TRAF6 Blue cells. LPS induced obvious accumulation of TRAF6 accompanied with higher association kinetics compared to the blank treatment group (Figure 5E, Video S1 and Video S2). The recovery kinetics of TRAF6 accumulation were analyzed by plotting the intensity difference between the two areas over time. The recovery rates TRAF6 accumulation in LPS-induced GFP-TRAF6-Blue cells were much faster than those in the blank treatment group, indicating that LPS treatment had a significant influence on the accumulation of TRAF6 (Figure 5E). Moreover, in time-lapse microscopy experiments, LPS-induced aggregation of TRAF6 showed droplet-like behavior, and underwent frequent fusion and occasional fission events, indicating that TRAF6 undergoes phase separation in cells during LPS treatment (Figure 5F, Video S3 and Video S4). Subsequently, we assessed the relative importance of the N-terminal versus the C-terminal of TRAF6 in phase-separated droplet assembly. We generated GFP-TRAF6-N-Terminal-Blue cell and GFP-TRAF6-C-Terminal-Blue cell, respectively, and investigated TRAF6 accumulation by immunofluorescent microscopy. Like the full-length TRAF6, we observed droplet-like accumulation in GFP-TRAF6-N-Terminal-Blue cells, but not in GFP-TRAF6-C-Terminal-Blue cells, during LPS stimulation (Figure 5G and Figure S4A), indicating that the N-terminal of TRAF6 plays a unique role in droplet-like accumulation.

To verify the phase separation capacity of TRAF6, we examined TRAF6 droplet assembly *in vitro* using purified proteins. Considering that the N-terminal of TRAF6 also showed droplet assembly as observed in GFP-TRAF6-N-Terminal-Blue cells, we purified and utilized the GFP-tagged N-terminal TRAF6 protein (1-347aa) for the subsequent studies. GFP-TRAF6-N-Terminal proteins *in vitro* formed liquid-like droplets that had high mobility, but were vulnerable to hexanediol (HEX) treatment, a molecule known to disturb hydrophobic interaction-induced phase separation assemblies, and high salt (Figure 5H-I and Figure S4B). In addition, GFP-TRAF6-N-Terminal proteins formed gel-like fibers with PEG treatment (Figure 5H). Collectively, these results demonstrated that TRAF6 undergoes phase separation *in vivo* and *in vitro* during LPS stimulation.

**Figure 6:**
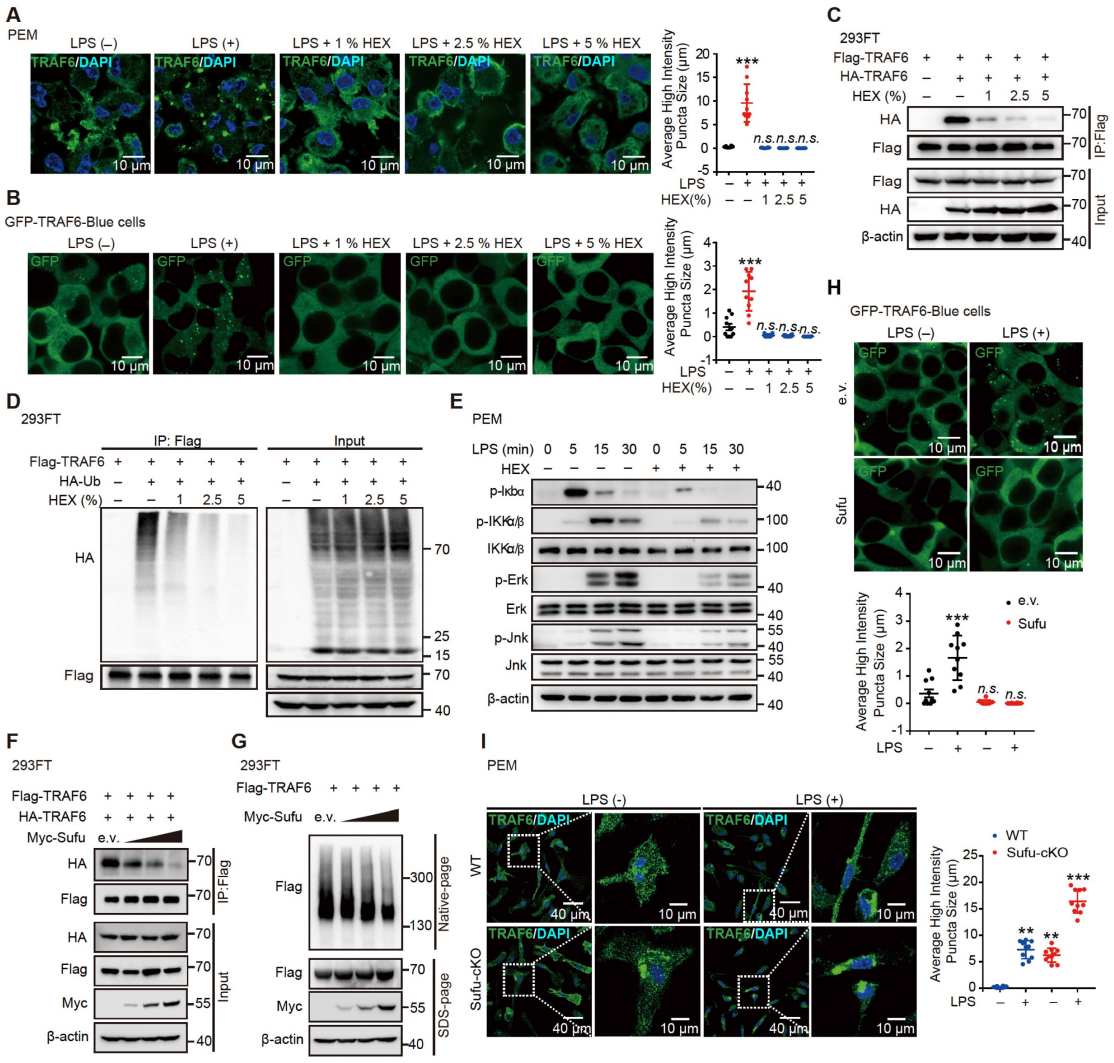
** Sufu represses TRAF6 aggregation and decreased droplet mobility.** (**A**) Representative images of TRAF6 (Green) at various times in PEMs after challenged with LPS (100 ng/mL) plus indicated concentrations of HEX. DAPI (Blue) was used for nuclear staining. Scale bar, 10 μm. (**B**) Representative images of GFP (Green) at various time points after challenge with LPS (100 ng/mL) and indicated concentrations of HEX in GFP-TRAF6-Blue cells. Scale bar, 10 μm. (**C**) Co-IP of Flag-tagged TRAF6 and HA-tagged TRAF6 in 293FT cells treated with indicated HEX, assessed by immunoblot analysis with anti-Flag or anti-HA after immunoprecipitation with anti-Flag. (**D**) Ubiquitination of TRAF6 in 293FT cells transduced with HA-ubiquitin (HA-Ub) and Flag-TRAF6 with or without indicated HEX treatment. (**E**) Immunoblot analysis of phosphorylated (p-) and total IKKα/β, Erk and Jnk, as well as p-Iκbα, and β-actin (loading control), lysates of PEMs derived from WT or Sufu-cKO mice and challenged with LPS (100 ng/mL) and indicated concentrations of HEX for various times. (**F**) Co-IP of Flag-tagged TRAF6 and HA-tagged TRAF6 in 293 FT cells transfected with Flag-tagged and HA-tagged TRAF6, plus either empty vector or increasing concentrations of Myc-tagged Sufu. Self-association of TRAF6 was assessed by immunoblot analysis with anti-HA or anti-Flag primary antibodies after immunoprecipitation with anti-Flag antibody. (**G**) Self-association of TRAF6 in 293FT cells transfected with Flag-tagged TRAF6, plus either empty vector or increasing concentrations (wedge) of Myc-tagged Sufu, then analyzed by immunoblot analysis with anti-Flag primary antibody in Native-PAGE. (**H**) Representative images of GFP (Green) in GFP-TRAF6-Blue cells transfected with either empty or Sufu-expressing vector, with or without LPS challenge (100 ng/mL). Scale bar, 10 μm. (**I**) **Left**, Representative images of TRAF6 in PEMs from WT and Sufu-cKO mice with or without LPS challenge (100 ng/mL). Scale bar, 40 μm. Right, magnification of the boxed region. Scale bar, 10 μm.

To illustrate that the LPS-induced phase separation and droplet formation of TRAF6 is important for its function, we treated LPS-induced cells with or without HEX (1,6-hexanediol) to disrupt phase separation of TRAF6 and then examine its ubiquitination and subsequent activation 54. Opposed to the effect of LPS stimulation, HEX treatment strongly diminished the assembly of droplet formation of TRAF6 in PEM (Figure 6A and Figure S5A) and GFP-TRAF6-Blue cells (Figure 6B and Figure S5B) in a dose-dependent manner. Consistent with these results, a Co-IP assay in 293FT cells transfected with Flag-tagged TRAF6 and HA-tagged TRAF6 showed abolished self-association of TRAF6 in response to HEX treatment (Figure 6C). Meanwhile, we transfected 293FT cells with HA-tagged ubiquitin (HA-Ub) and Flag-tagged TRAF6 with or without HEX treatment, then performed immunoprecipitation experiments with anti-FLAG antibody. Cells transfected with HA-Ub, Flag-tagged TRAF6 in the absence of HEX showed high levels of ubiquitination; however, such ubiquitination was substantially attenuated in the presence of HEX in a dose-dependent manner (Figure 6D). Furthermore, HEX treatment impeded LPS signaling transduction as shown by decreased phosphorylation of Iκbα and downstream kinases (IKKα/β, Jnk and Erk) (Figure 6E). Together, these results demonstrated that disrupting phase separation of TRAF6 abolished its autoubiquitination and subsequent activation.

### Sufu represses phase droplet formation of TRAF6

Since the N-terminal of TRAF6 contributed to its phase separation and this region was essential to the interaction with Sufu, we speculated that Sufu might function through interfering phase-droplet formation of TRAF6, thereby restraining it signaling activity. To test this possibility, we first transfected 293FT cells with Flag-tagged TRAF6 and HA-tagged TRAF6, as well as empty vector or increasing doses of Sufu. Co-IP results showed stronger homo-association of TRAF6 in the empty vector group, but such homo-association was progressively decreased along increased expression of Sufu (Figure 6F). Consistently, we transfected 293FT cells with Flag-tagged TRAF6 and increasing doses of Sufu, followed by Native-PAGE to detect homo-associated TRAF6 as polymers. We found that Sufu disturbed the homo-oligomerization of TRAF6 in a dose-dependent manner (Figure 6G). Next, we transfected GFP-TRAF6 Blue cells with empty vector or Myc-Sufu. We found that LPS treatment induced apparent aggregation of TRAF6, while expression of Sufu largely eliminated such aggregation (Figure 6H). In addition, we observed similar droplet-like accumulation in GFP-TRAF6-N-Terminal-Blue cell, while such droplet formation was absent in cells expressing Sufu (Figure S5C). Moreover, the purified TRAF6 N-terminus protein showed the ability to condensate into droplets in size of micrometer scale; and this ability was abrogated by addition of the purified Sufu protein in a dose-dependent manner (Figure S5D). Subsequent FRAP assay also revealed that overexpression of Sufu sharply inhibited the liquid-like dynamic movement of TRAF6 droplets, as shown by the more immobile fraction (Figure S5E, Video S5 and Video S6), indicating that Sufu directly inhibits the assembly of TRAF6 droplets.

To further examine Sufu regulated-TRAF6 phase separation *in vivo*, we obtained PEMs from WT and Sufu-cKO mice, followed by LPS treatment, respectively. LPS stimulation induced significant TRAF6 droplets formation in the PEMs from WT mice. Importantly, TRAF6 spontaneously formed phase-separated droplets in PEMs from Sufu-cKO mice even in the absence of LPS, and LPS stimulation further enlarged the average size of these droplets (Figure 6I). Taken together, these results demonstrated that Sufu directly represses TRAF6 phase-separated droplets formation of TRAF6, thereby limiting TRAF6-mediated signaling activity during inflammatory responses.

### Knockdown of TRAF6 rescues augmented inflammatory phenotype of Sufu-cKO mice

**Figure 7:**
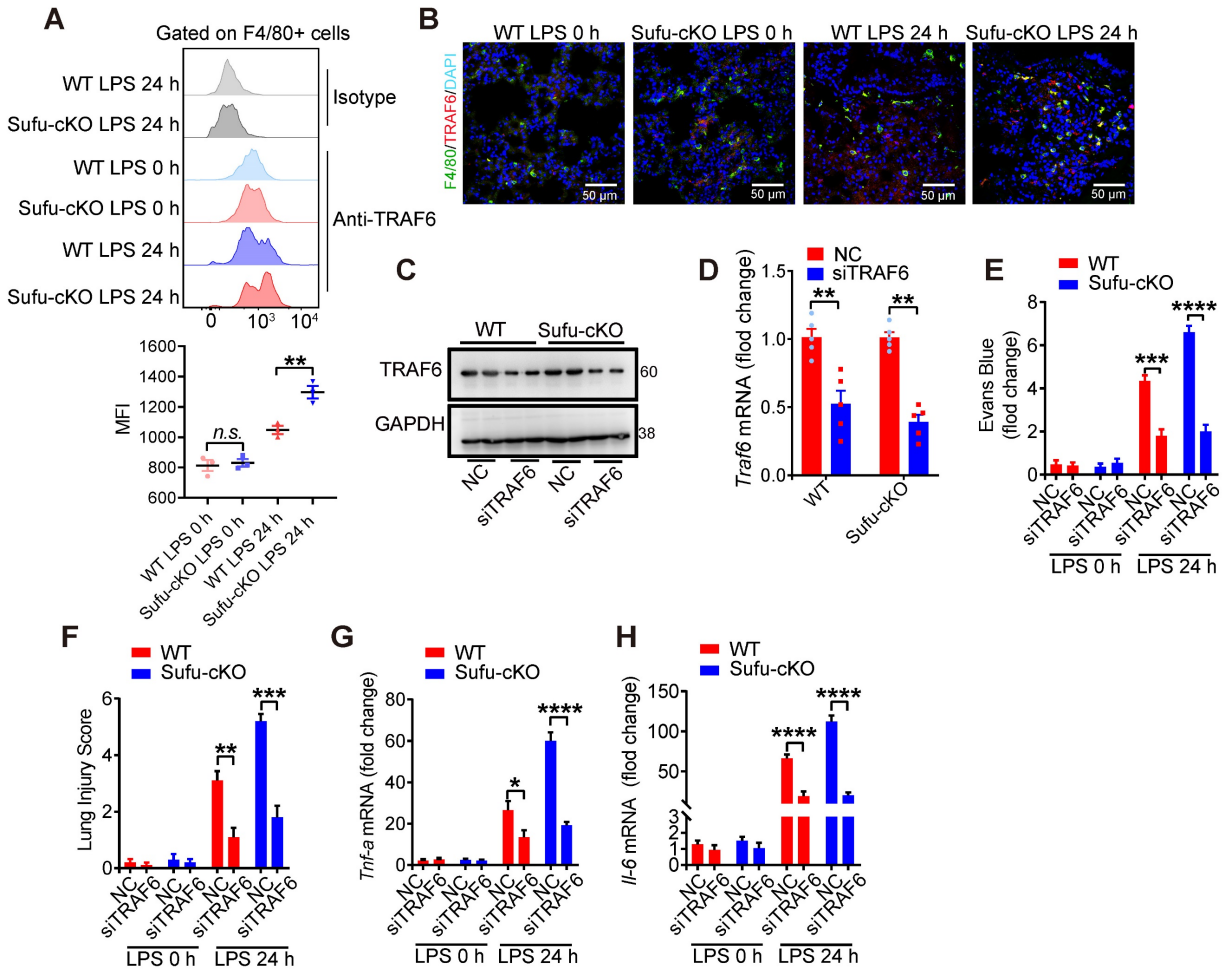
** Knockdown of TRAF6 prevents LPS-induced lung inflammation in Sufu-cKO mice.** (**A**) Flow cytometric analysis for TRAF6 in lung macrophages from WT and Sufu-cKO mice 24 h post LPS challenge (5 mg/kg). (**B**) Immunostaining for TRAF6 (Red) and F4/80 (Green) in lung section from WT and Sufu-cKO mice 24 h post LPS challenge (5 mg/kg). DAPI (Blue) was used for nuclear staining. Scale bar, 50 μm. (**C** and **D**) Sufu-cKO mice were transfected with NC or TRAF6 siRNA through tail vein injection for 48 h, then challenged with LPS (100 ng/mL) for 24 h. Representative Western blot (**C**) and qRT-PCR (**D**) analysis showing TRAF6 knockdown in lung. Means ± SEM (n = 5). **p < 0.01. (**E**) Sufu-cKO and WT mice were intraperitoneally injected with PBS or LPS (5 mg/kg) for 23 h, then injected with Evans blue dye (30 mg/kg body weight) for an additional 40 min before mice were killed and lung tissues harvested. Means ± SEM (n = 5). ***p < 0.001. ****p < 0.0001. (**F**) Quantification of histopathological lung injury scores. Means ± SEM (n = 5). **p < 0.01. ***p < 0.001. (**G and H**) Quantitative analysis of *Tnfα* (**G**) and *Il-6* (**H**) mRNA levels in lung by qRT-PCR. Means ± SEM (n= 5). *p < 0.05. ****p < 0.0001.

To identify whether a specific macrophage subset manifested TRAF6 functional suppression mediated by Sufu to prevent lung injury, we first evaluated the degree of protein aggregation in vivo by flow cytometric analysis. Flow cytometric analysis showed accumulation of TRAF6 protein in the lung macrophages from LPS-induced septic shock model of Sufu-cKO mice, compared to those from WT mice (Figure 7A-B). To further address whether TRAF6 expression was required and sufficient for sepsis-induced lung inflammation in Sufu-cKO mice, we designed a TRAF6-specific small interfering RNA (siRNA) and transfected Sufu-cKO mice by liposome incorporation to knockdown endogenous TRAF6 expression. TRAF6 protein and mRNA levels were substantially reduced in TRAF6-specific siRNA-transfected Sufu-cKO mice compared to those transfected with non-targeting control siRNA (Figure 7C-D). To further examine the effect of TRAF6 depletion on pulmonary permeability and disruption of the pulmonary vascular barrier in Sufu-cKO mice, we injected Evans blue dye (30 mg/kg body weight) in both Sufu-cKO and WT mice challenged with LPS, then measured the extravasation of Evans blue in the lung. We found that knockdown of TRAF6 in Sufu-cKO mice rescued LPS-induced pulmonary vascular hyperpermeability (Figure 7E). Moreover, knockdown of TRAF6 alleviated lung injury caused by augmented inflammation in Sufu-cKO Mice (Figure 7F). Knockdown of TRAF6 also resulted in decreased mRNA expression of Tnfα and Il-6 in Sufu-cKO mice induced with LPS (Figure 7G-H), suggesting that Sufu suppressed lung inflammation during sepsis via interfering TRAF6 function.

## Discussion

As a key signal transducer for multiple pathways, the E3 ubiquitin ligase TRAF6 plays a pivotal role in innate and adaptive immunity. Tight regulation of TRAF6 signaling activity is crucial for maintaining immunological homeostasis. Our study uncovered for the first time that TRAF6 undergoes phase separation to form droplets upon LPS signaling, and further identified that Sufu, a member of the Hh signaling pathway, acts as a “molecular break” that limits TLR-related inflammatory responses through disrupting the phase separation and droplet formation of TRAF6.

The role of Hh signaling is well established in cancer and lipid metabolism-related disease such as fatty liver disease and atherosclerosis. Our lab recently reported that Hh signaling pathway induces a previously undefined long non-coding RNA (Hilnc, Hh signaling-induced long non-coding RNA), which controls hepatic lipid metabolism 55. Hh signaling pathway is known to function in adult tissue repair, macrophages polarization, and inflammatory response, while inflammatory factors can also regulate Hh signaling pathway in canonical or non-canonical manners. Sepsis is associated with acute organ dysfunction and a high risk of death. Although multiple pathways involved in inflammation, tissue damage and aberrant healing process during sepsis have been identified there remain unmet needs for the development of new therapeutic strategies to prevent reoccurrence of infection and organ injury. We found that the expression of Sufu dynamically responds to TLR signaling, including LPS treatment, CLP, poly (I:C), R-848 and ODN1585. Given that TRAF6 is employed in multiple innate signaling pathways, Sufu might generally respond to and negatively regulate TRAF6-related signaling. In the classical Hh signaling pathway, activation of Smo uncoupled the binding and inhibition of Gli1 by Sufu, and Smo activation promoted phosphorylation of Sufu at Ser342 and Ser346 by PKA and GSK3β, thereby promoting Sufu ubiquitination and degradation 56. Thus, it is conceivable that Hh signal members may play an important role in innate immune response. In this study, we found that the transcription level of *Ptch1* showed little response to LPS stimulation, while *Smo* mRNA level displayed an opposite fluctuation to that of Sufu. Previous research has demonstrated that the Hh signaling pathway positively regulates the expression of Traf6 through the Smo/Gli2 axis and stabilizes TRAF6 protein, ultimately leading to the mediation of osteoclast differentiation 57. Therefore, we examined whether the regulation of TRAF6 by Sufu was inhibited by Smo, and found that the interaction between Sufu and TRAF6 was inhibited by Smo (data not shown). This is consistent with the Hh signaling pathway in which Smo inhibited the function of Sufu but does not interact with Sufu.

The functional importance of K63-linked autoubiquitination of TRAF6 has been studied extensively 31, 47, 58-60. Various negative regulators have been identified by studies targeting TRAF6 autoubiquitination. Previous research has demonstrated that the activation of TRAF6 is dependent on the K63 auto-polyubiquitination process, which can be inhibited by deubiquitinates such as A20, CYLD, MCPIP1, USP4, or USP2a 30, 61-66**.** A20 is a ubiquitin-editing enzyme that could dampen TLR signaling by targeting TRAF6 30, 31. In response to TLR signaling, A20 is expressed and associates with TRAF6 to remove its K63-linked ubiquitin chains. USP2a is a protein that interacts with MALT1 and TRAF6, and its role is to remove K63-linked polyubiquitin chains from TRAF6. This function is crucial in TCR signaling, and it is achieved by deSUMOylating TRAF6 and mediating TRAF6-MALT1 interaction 66. In the presence of IL-33, TRAF6 is recruited to IL-33R, which results in increased K27-linked polyubiquitination of IL-33RK511, promoting the stability of IL-33R by inhibiting its autophagic degradation. Ultimately, this leads to the activation of IL-33R-mediated signaling 67. Our findings demonstrated that Sufu helped to prevent excessive inflammatory responses by directly interacting with TRAF6, interfering its homo-oligomeric association. Under immune homeostasis, Sufu may prevent spontaneous TRAF6 activation and downstream inflammatory signaling through this mechanism. Upon LPS stimulation, acute downregulation of Sufu allowed TRAF6 to oligomerize and fully activate. As the inflammatory response progressed, the recovered Sufu level may act to terminate TRAF activity and the inflammatory action. In this context, Sufu might act as a checkpoint for TRAF6-mediated inflammatory response to maintain immune homeostasis through a distinct mechanism from that of A20. The phase separation of TRAF6 triggered by LPS stimulation and that Sufu restrained such action of TRAF6 was first observed during our investigation. It has been revealed that the N-terminal domain of TRAF6 is essential for its dimerization and the C-terminal permits TRAF6 trimerization respectively. This structural feature facilitates TRAF6 as both a signal transducer and an adapter protein in the TLR pathway. In this scenario, phase separation acts by providing proper concentration and mobility of TRAF6 to achieve a rapid inflammatory signaling transduction. Through further mechanistic studies, we found that Sufu can directly interact with TRAF6 and this interaction is modulated by LPS stimulation. Because Sufu expression rapidly diminished in response to LPS stimulation, we proposed that the varying levels of Sufu protein allowed rapid control of TRAF6 phase separation and droplet formation, therefore limiting TRAF6-mediated signaling to a rapid but confined burst. Recent work reported that the STING also undergoes phase separation 38. Given that TRAF6 could interact with STING, we cannot rule out the possibility that TRAF6 may undergo co-phase separation concerted with other key signaling molecules such as STING. Importantly, a recently published study showed that multivalent interactions between NEMO and polyUb (generated by TRAF6) led to NEMO phase separation and IKK activation in NF-κB signaling 68. The authors showed that NEMO condensates contained TRAF6 and the TAK1 kinase complex, supporting our current study which not only revealed phase separation of TRAF6 but also implicated Sufu as key regulator of this process.

Our findings, that Sufu was downregulated in patients with sepsis and that it protected mice from septic shock, implied a novel role of Sufu in the pathogenesis of certain infectious diseases. Moreover, our data indicated that the role of Sufu in limiting inflammatory damage is largely dependent on macrophages. Macrophages have been tightly linked to tumorigenesis with dysregulated function of the immune system. In this context, we speculate that Sufu might also be important in modulating tumor-related inflammatory responses through macrophages. In Sufu-cKO mice, we demonstrated that TRAF6 expression was required for sepsis-induced lung inflammation. TRAF6 not only plays an important role in the TLR-mediated innate immune response, but also participates in the RIG-mediated antiviral signaling pathway. Whether Sufu can regulate other TRAF6-mediated intrinsic immune pathways by affecting TRAF6 phase separation needs further investigation. Nevertheless, the regulatory network for TRAF6 appears to be more complicated than previously appreciated, and multiple surveillance mechanisms are clearly in place to ensure its proper signaling.

In conclusion, we report that upon LPS signaling, TRAF6 undergoes phase separation - a previously undefined phenomenon. We further showed that such phase separation of TRAF6 is regulated by Sufu, exemplifying a novel regulatory mechanism for innate immunity. These results indicated that Sufu plays an important role in the regulation of the inflammatory response and may serve as a potential target for new therapeutic interventions against sepsis-associated diseases.

## Supplementary Material

Supplementary figures, table, video legends.

Supplementary video 1.

Supplementary video 2.

Supplementary video 3.

Supplementary video 4.

Supplementary video 5.

Supplementary video 6.
