# Supplementary material for: Sufu limits sepsis-induced lung inflammation via regulating phase separation of TRAF6
Source: Theranostics. 2023 Jun 26;13(11):3761–80. doi: 10.7150/thno.83676 (PMC10334838; doi:10.7150/thno.83676)
Supplement: Supplementary file 1 — Supplementary figures, table, video legends. [file thnov13p3761s1.pdf]

Supplementary Material

Sufu limits sepsis-induced lung inflammation via regulating phase separation of TRAF6

Yehua Li[#], Jiayin Peng[#,*], Yuanxin Xia, Chenyu Pan, Yu Li, Weijie Gu, Jia Wang, Chaoxiong Wang, Yuang Wang, Jiawen Song, Xuan Zhou, Liya Ma, Yiao Jiang, Biao Liu, Qiongni Feng, Wenjia Wang, Shi Jiao, Liwei An, Dianfan Li, Zhaocai Zhou[*], Yun Zhao[*]

The PDF file includes:

Figures S1 to S5

Videos S1 to S6

Table S1

Supplementary Figure 1

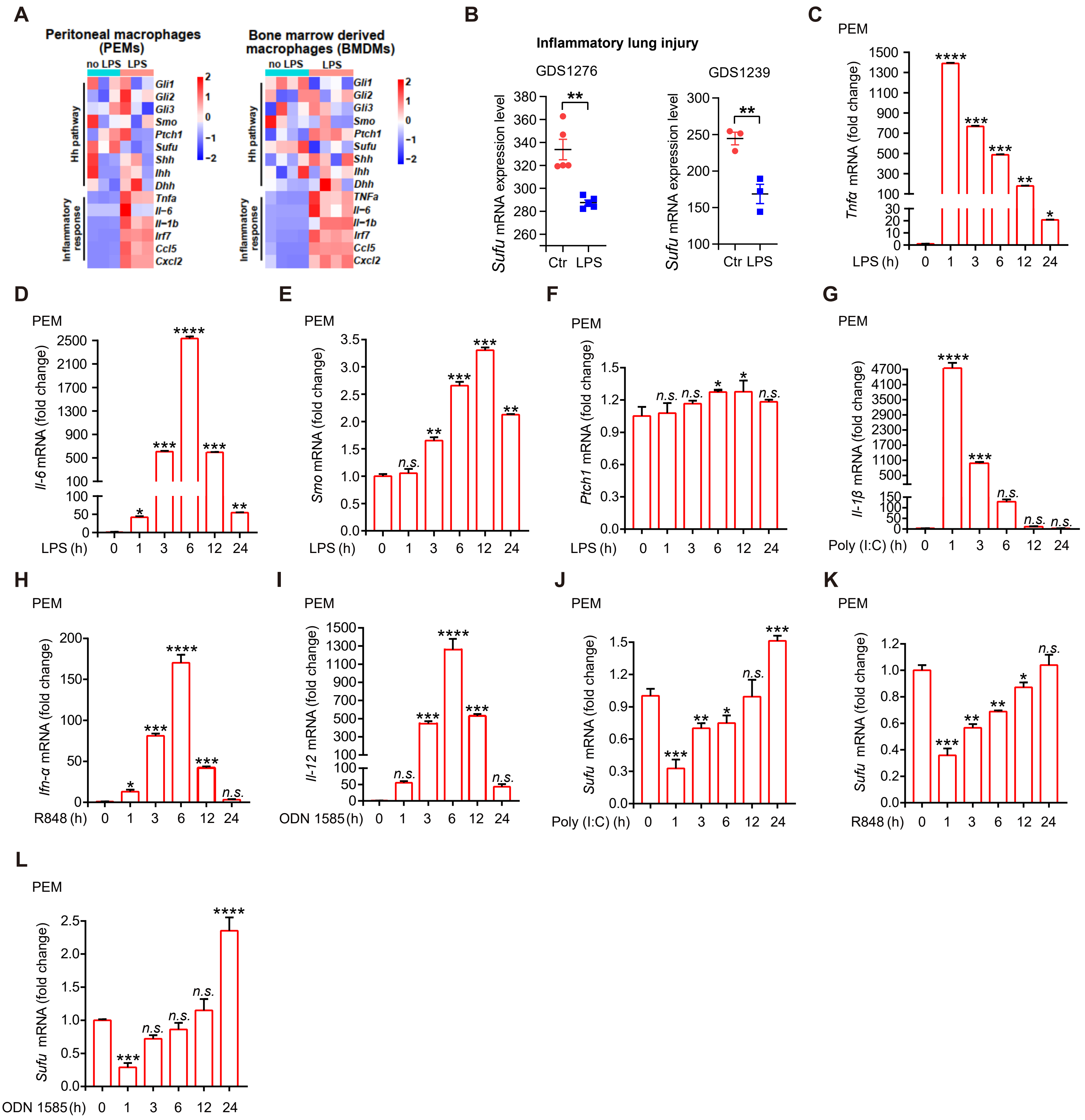

Figure S1. Fluctuation of Sufu in response to inflammatory stimuli. （**A**） Relative mRNA levels of genes with or without LPS treatment in GEO databases (PEMs: GSE2002 and BMDMs: GSE53986). (**B**) Scatter plot for *Sufu* mRNA levels from data sets GDS1276 and GDS1239. Means ± SEM. ** $p < 0.01$. (**C** and **D**) Quantitative analysis of *Tnfα* (**C**) and *Il-6* (**D**) mRNA levels in PEMs after LPS (100 ng/mL) challenge at indicated times by qRT-PCR. Results are presented relative to those of the control gene *Gapdh* and normalized to 0 h. (**E** and **F**) Quantitative analysis of *Smo* (**E**) and *Ptch1* (**F**) mRNA levels in PEMs after LPS (100 ng/mL) challenge at indicated times by qRT-PCR. Results are presented relative to those of the control gene *Gapdh* and normalized to 0 h. (**G**) The expression of *Il-1β* mRNA level in PEMs after challenge with Poly (I:C) for various times (horizontal axis), results are presented relative to those of the control gene *Gapdh* and normalized to 0 h. (**H**) The expression of *Ifn-α* mRNA level in PEMs after challenge with R848 for various times (horizontal axis), results are presented relative to those of the control gene *Gapdh* and normalized to 0 h. (**I**) The expression of *Il-12* mRNA level in PEMs after challenge with ODN 1585 for various times (horizontal axis), results are presented relative to those of the control gene *Gapdh* and normalized to 0 h.(**J-L**) Quantitative analysis of *Sufu* mRNA levels in PEMs after Poly (I:C) (**J**), R848 (**K**) or ODN 1585 (**L**) challenge at indicated times by qRT-PCR. Results are presented relative to those of the control gene *Gapdh* and normalized to 0 h. Means ± SEM. * $p < 0.5$. ** $p < 0.01$. ***$p < 0.001$. ****$p < 0.0001$. ns, no significance.

Supplementary Figure 2

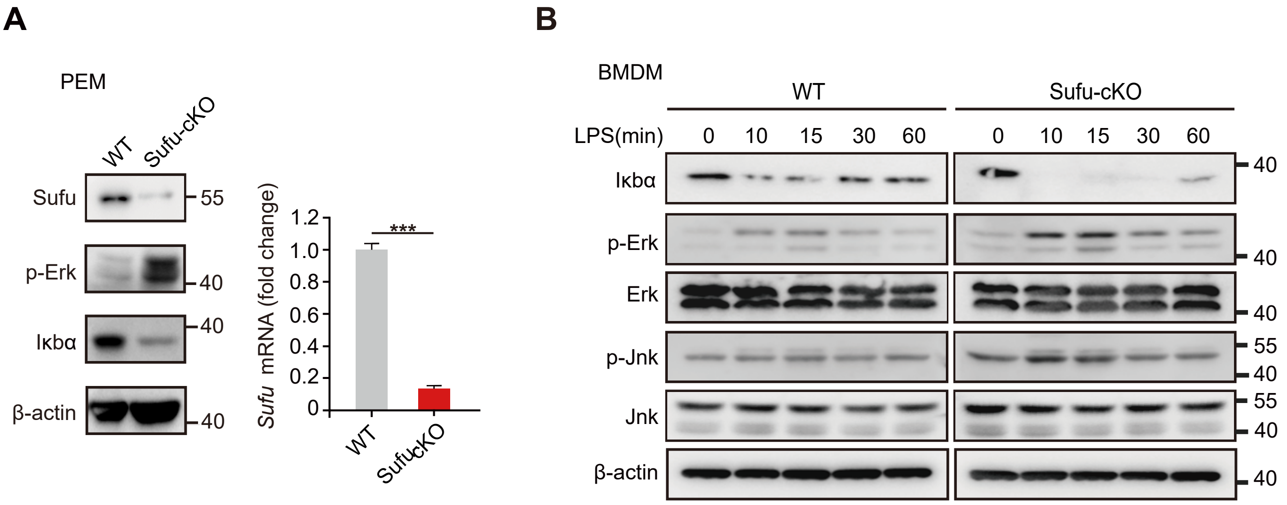

Figure S2. Knockout of Sufu promotes inflammatory responses.

(**A**) **Left**, immunoblot analysis of Sufu, p-Erk, Iκbα, and β-actin (loading control), lysates of PEMs derived from WT and Sufu-cKO mice. **Right**, *Sufu* mRNA change of PEMs derived from WT and Sufu-cKO mice. Means ± SEM. ***$p < 0.001$. (**B**) Immunoblot analysis of phosphorylated (p-) and total IKKα/β, Erk, Jnk, p-Iκbα, and β-actin (loading control) in lysates of BMDMs derived from WT or Sufu-cKO mice challenged with LPS (100 ng/mL) and isolated at indicated time points post challenge.

Supplementary Figure 3

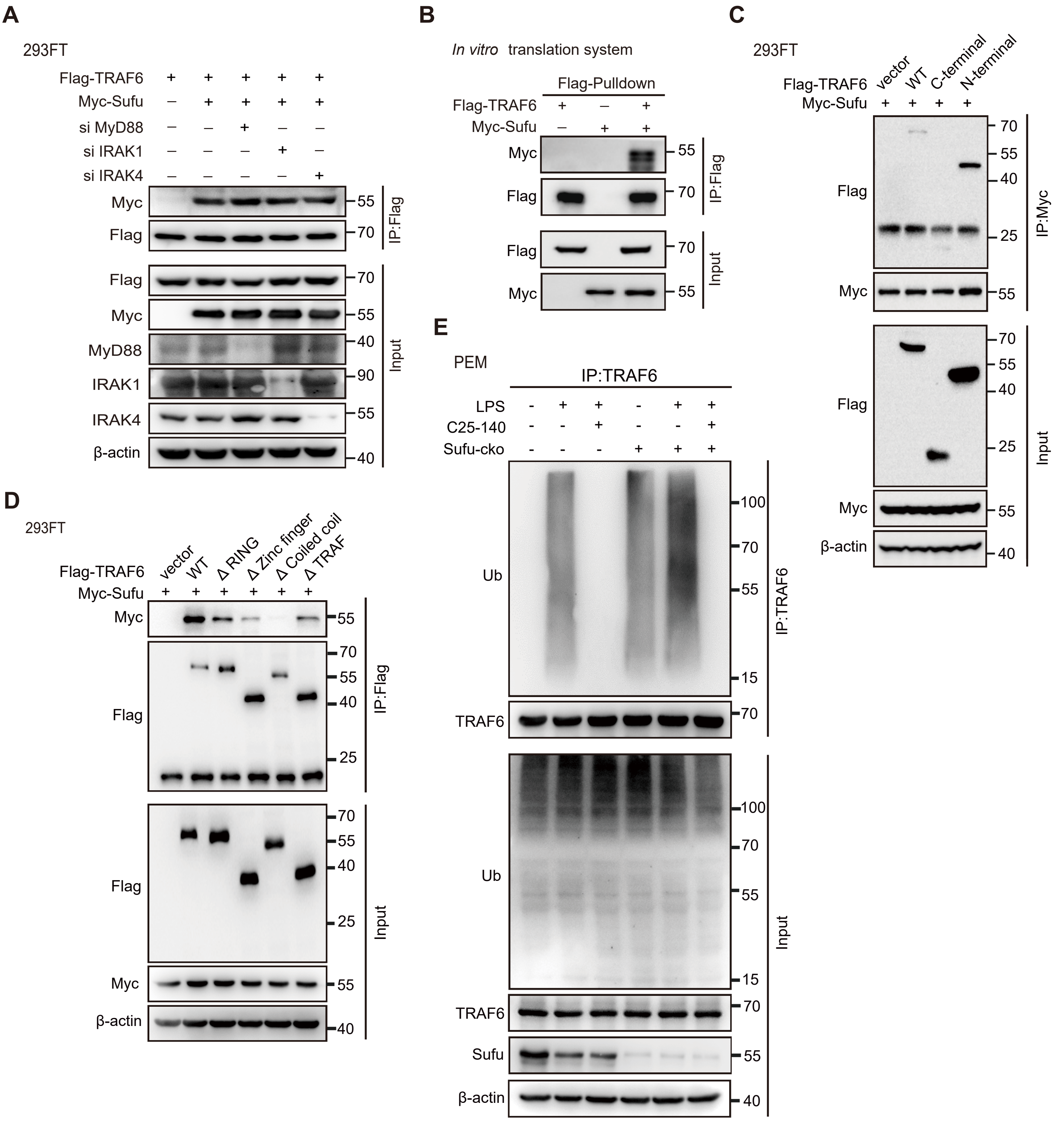

Figure S3. Interaction of Sufu and TRAF6.

(**A**) Co-immunoprecipitation of Sufu with TRAF6 transfected with siRNA of Myd88, IRAK1 or IRAK4, respectively. (**B**) *In vitro* pull-down assay of Myc-tagged Sufu and Flag-tagged TRAF6, assessed by immunoblot analysis with anti-Myc or anti-Flag after Co-IP with anti-Flag or not. (**C**) Co- IP of Sufu with TRAF6 truncations in lysates of 293FT cells transfected with vector encoding Myc-tagged Sufu and Flag-tagged TRAF6 truncations, assessed by immunoblot analysis with anti-Flag, anti-Myc after Co-IP with anti-Myc or not. (**D**) IP mapping of specific domains responsible for TRAF6 interaction with Sufu. Data are representative of three independent experiments with similar results. Of note: TRAF6△RING, delete amino acids 70-108; TRAF6△Zinc finger, delete amino acids 149-259; TRAF6△Coiled coil, delete amino acids 288-347; TRAF6 △ TRAF, delete amino acids 355-482. (**E**) Ubiquitination of endogenous TRAF6 in PEM cells from WT and Sufu-cKO mice with or without LPS challenge (100 ng/mL) after C25-140 treatment (20 μM, 2 h).

Figure S4. Phase separation of TRAF6 during LPS stimulation.

(**A**) Representative images of GFP (Green) with or without LPS stimulation in GFP-TRAF6-C-terminal-Blue cells. Scale bar, 20 μm (Above) or 5 μm (Bottom). (**B**) GFP-TRAF6-N-Terminal phase separation with different salt concentrations. Scale bar, 5 μm (Above) or 10 μm (Bottom).

Supplementary Figure 4

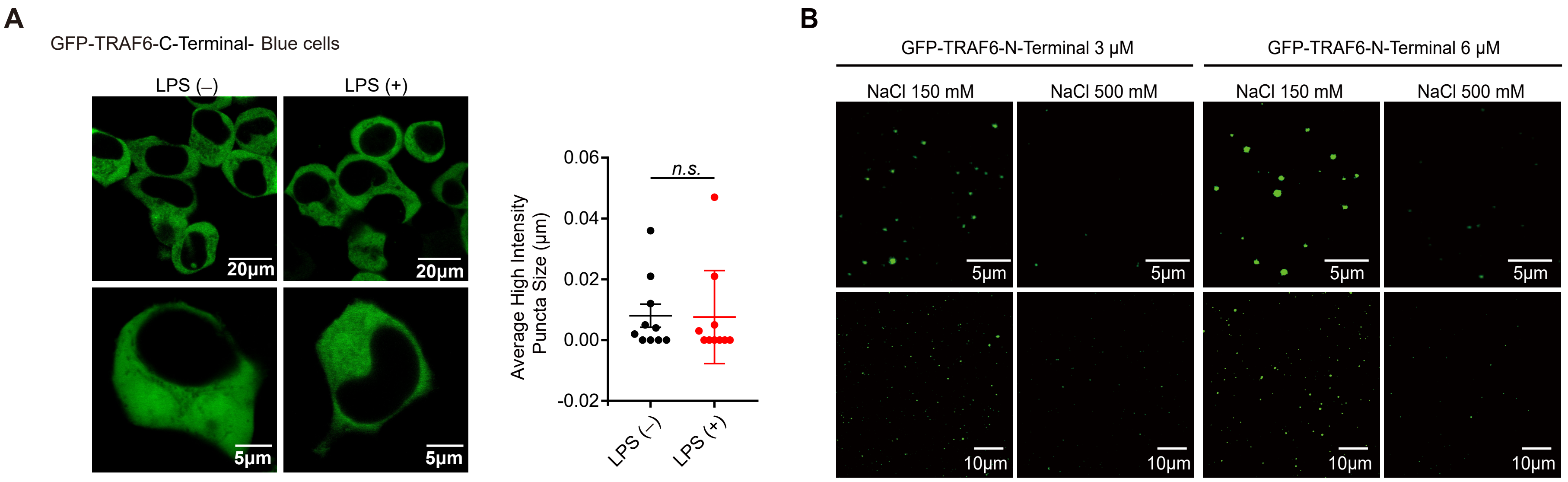

Figure S5. Sufu represses phase droplet formation of TRAF6

(**A**) Representative images of TRAF6 (Green) at various times in PEMs after challenged with LPS (100 ng/mL) plus indicated concentrations of HEX. DAPI (Blue) was used for nuclear staining. Scale bar, 10 μm. (**B**) Representative images of GFP (Green) at various time points after challenge with LPS (100 ng/mL) and indicated concentrations of HEX in GFP-TRAF6-Blue cells. Scale bar, 10 μm. (**C**) Representative images of GFP (Green) in GFP-TRAF6-N-terminal-Blue cells transfected with either empty or Sufu-expressing vector, with or without LPS challenge (100 ng/mL). Scale bar, 20 μm (left), 5 μm (right). (**D**) GFP-TRAF6-N-Terminal proteins liquid-like droplets formation after transfected with increasing concentrations of Sufu purified protein. Scale bar, 5 μm (above) or 10 μm (bottom). (**E**) Left, Representative fluorescent recovery after photobleaching (FRAP) images of GFP-TRAF6-Blue cells transfected with either empty vector or increasing concentrations of Sufu after challenge with LPS. Scale bar, 5 μm. White circles indicate bleached regions. For examples of fluorescence bleaching, see Video S5 and S6. Right, Kinetics of TRAF6 recovery in GFP-TRAF6-Blue cells.

Supplementary Figure 5

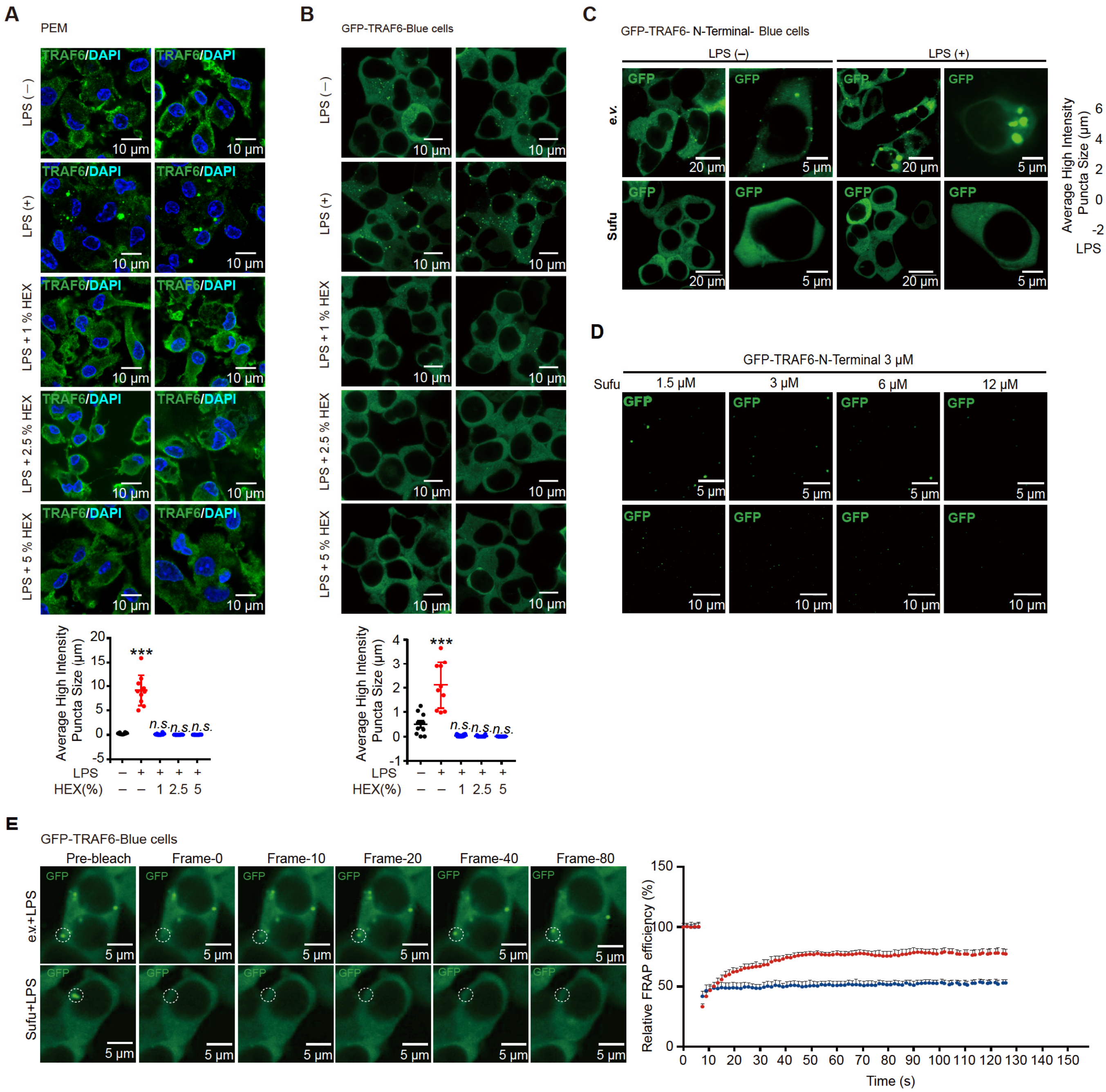

Supplementary Videos

Video S1 and Video S2

GFP-TRAF6-Blue cells were cultured on 29 mm No.1.5 glass-bottomed dishes 24 h before imaging. Cells were treated with or without LPS 30 min followed by imaging with Leica TSC SP8 STED 3X. Photobleaching and subsequent image acquisition (5 prebleached images, and a sequence of post-bleach images at 80 images every 1.5 s). Data were calculated and fitted in a one-phase exponential association curve by GraphPad Prism 7. Arrows indicated the TRAF6 protein photobleached. Videos 1-2 show fluorescence recovery of TRAF6 in PBS-treated (Video S1) and LPS-treated (Video S2) GFP-TRAF6-Blue cells.

Video S3 and Video S4

To visualize fusion events of TRAF6 in living cell, GFP-TRAF6-Blue cells were cultured on 35 mm No. 1.5 glass-bottomed dishes. Cells were washed once with PBS and the medium was replaced by FluoroBrite DMEM (GIBCO) supplemented with 10% FBS and placed back in the incubator for 30 min LPS treatment followed by imaging with Leica TSC SP8 STED 3X. Arrows indicated fusion TRAF6 protein.

Video S5 and Video S6

Plasmid e.v. and Sufu were transfected in GFP-TRAF6-Blue cells 24 h before cultured on 29 mm no.1.5 glass-bottomed dishes. After overnight cultured, cells were treated with 30 min LPS treatment, followed by imaging with Leica TSC SP8 STED 3X. Photobleaching and subsequent image acquisition (5 prebleached images, and a sequence of post-bleach images at 80 images every 1.5 s). Arrows indicated the TRAF6 protein photobleached. Videos S5-6 show fluorescence recovery of TRAF6 in plasmid e.v. (Video S5) or Sufu-transfected (Video S6) GFP-TRAF6-Blue cells.

Supplementary Table

Table S1: List of primer sequences used for RT-qPCR

Gene	Pimer sequences (5'-3')
Tnfα	Fw -CCTGTAGCCCACGTCGTAG<br>Rv - GGGAGTAGACAAGGTACAACCC
Il6	Fw-TCTATACCACTTCACAAGTCGGA<br>Rv - GAATTGCCATTGCACAACTCTTT
TRAF6	Fw - AAAGCGAGAGATTCTTTCCCTG<br>Rv - ACTGGGGACAATTCACTAGAGC
Il-1β	Fw -GAAATGCCACCTTTTGACAGTG<br>Rv -TGGATGCTCTCATCAGGACAG
Ifn-β	Fw -TGGGTGGAATGAGACTATTGTTG<br>Rv -CTCCCACGTCAATCTTTCCTC
Il-12	Fw - GCTGCTGCGTTGAGAAGACA<br>Rv -CACAGGACGTGAGAAACATTGT
Smo	Fw - GGAGGCTACTTCCTCATCAG<br>Rv - GCTTGGCATAGCACATAGTC
Ptch1	Fw - ACTACCCGAATATCCAGCAC<br>Rv - ATCCTGAAGTCCTTGAAGCC
Sufu,	Fw- CGGACCCCTTGGACTATGTTA<br>Rv - CTTCAGACGAAACGTCAACTCA
Gapdh	Fw- TGAAGCAGGCATCTGAGGG<br>Rv -CGAAGGTGGAAGAGTGGGAG
